# Contrastive learning to fine-tune feature extraction models for the visual cortex

**DOI:** 10.1371/journal.pcbi.1014656

**Published:** 2026-08-17

**Authors:** Alex Mulrooney, Zhi Li, Austin J. Brockmeier

**Affiliations:** 1 Department of Electrical and Computer Engineering, University of Delaware, Newark, Delaware, United States of America; 2 Department of Computer and Information Sciences, University of Delaware, Newark, Delaware, United States of America; Weill Cornell Medicine, UNITED STATES OF AMERICA

## Abstract

Predicting the neural response to natural images in the visual cortex requires extracting relevant features from the images and relating those feature to the observed responses. In this work, we optimize the feature extraction in order to maximize the information shared between the image features and the neural response across voxels in a given region of interest (ROI) extracted from the BOLD signal measured by functional magnetic resonance imaging (fMRI). We adapt contrastive learning (CL) to fine-tune a convolutional neural network, which was pretrained for image classification, such that a mapping of a given image’s features are more similar to the corresponding fMRI response than to the responses to other images. We exploit the Natural Scenes Dataset as organized for the Algonauts Project, which contains the high-resolution fMRI responses of eight subjects to tens of thousands of naturalistic images. We show that CL fine-tuning creates feature extraction models that enable higher encoding accuracy in both early and higher visual ROIs as compared to the features from the pretrained network. Quantitatively, the performance is similar to a baseline approach that directly uses a regression loss at the output of the network to tune it for fMRI response encoding. We investigate inter-subject transfer of the CL fine-tuned models, including subjects from the Natural Object Dataset, another lower-resolution dataset with 9 subjects. We also pool subjects for fine-tuning, which further improves encoding performance in early ROIs. Finally, we examine the performance of the fine-tuned models on common image classification tasks, explore the landscape of ROI-specific models by applying dimensionality reduction on the Bhattacharya dissimilarity matrix created using the predictions on those tasks, and show that these landscapes match those based on representational similarity analysis. Finally, we generate images via Stable Diffusion based on vector-space prompts created by aligning the CL-tuned models embeddings for different ROIs, showing that generated images have similar embeddings to the original but that estimates of the intrinsic dimension are lower for generated versus original representations.

## Author summary

We propose a methodology for predicting the neural response in different regions of the visual cortex to natural images by fine-tuning a pretrained neural network for each region. We show that fine-tuning neural networks using either contrastive learning or to directly predict the neural response creates representations of the images more similar to the representations in the corresponding brain regions that they were fine-tuned on, as evidenced by the improvements in neural response prediction, which are further improved by pooling together the data from the same brain region in different subjects to fine-tune one model. Furthermore, we show clear anatomical organization among brain regions when assessing the similarity of predictions of the models tuned on different brain regions on downstream image classification tasks, which match results using representational similarity analysis. Region-specific tuned models can be used for “in-silico” investigations of brain function, and we present a simple demonstration towards this end using image generation.

## Introduction

Recent advances in computing power, large accessible datasets, and machine learning (especially deep learning) methods allow for new computational models of the brain. Modern approaches enable more powerful decoding of stimuli from neural responses [1] and prediction of neural responses from stimuli via encoding models [2]. In visual neuroscience, the Natural Scenes Dataset [3] provides a large dataset of brain responses to colored natural imagery for eight subjects measured by 7T fMRI BOLD responses (1.8mm-wide voxels, 1.6s sampling interval). The high resolution and high number of trials (close to 10,000 per subject) coupled with the complexity and breadth of imagery allows for more fine-grained modeling using deep learning. To this end, we propose a novel neural encoding approach that adapts a contrastive learning (CL) loss function [4,5] to fine-tune a pretrained convolutional neural network (CNN) to produce feature representations of stimulus images that better align with the voxel-wise neural activity in specific regions of interest (ROIs) in the visual cortex. We fine tune a different instance of the CNN for each ROI, with different versions for each subject along with a pooled subject model.

Our method differs from previous approaches to fMRI encoding [6,7] that use predetermined feature maps to build linear models for individual voxels. We still use linear models for predicting the activity of individual voxels from the fine-tuned image features, but we fine-tune CNNs to act as ROI-specific non-linear feature extraction models for each subject and ROI, with ROIs spanning the entire visual cortex. Our approach is motivated by the fact that different ROIs in the visual cortex encode different visual characteristics. Once fine-tuned using CL, the set of ROI- and subject-specific CNNs are used for encoding and are analyzed in terms of the accuracy and similarity of their predictions on downstream image classification tasks, which can be visualized as a landscape of models [8]. In particular, we fine-tune a pretrained CNN, specifically AlexNet trained on ImageNet [9], a relatively simple deep learning architecture, optimizing both the convolutional and fully-connected layers. However, the CL approach and the model landscape are general approaches and can be applied to other CNNs and feature extraction models beyond CNNs, such as Vision Transformers [10], and can also be applied to other stimulus modalities such as speech and sound.

For comparison, we use as a baseline the image features obtained from the pretrained, but untuned, AlexNet, and as another baseline we use an approach that fine-tunes the CNN using a regression loss at its output to predict the voxel activations in the ROI. With CL-based tuning, we note consistent improvements in encoding performance for early ROIs compared to the untuned AlexNet baseline. This result holds for another lower-resolution fMRI dataset, the Natural Object Dataset (NOD) [11]. Our contributions are as follows:

- We introduce a novel neural encoding approach that uses CL to fine-tune a CNN for ROI-specific feature extraction.
- We show that models trained with CL enable significant improvements in encoding performance in terms of average correlation between predicted responses and actual responses on held-out test images versus the untuned baseline across ROIs, especially in ones early in the visual processing stream (V1, V2, V3, and hV4). Regression-tuning also improves the encoding performance, achieving higher average correlation but with a lower number of voxels improved over the baseline.
- We use 9 subjects from the Natural Object Dataset [12], which uses atlas-based ROIs, to validate the CL-tuned feature extraction models for early visual ROIs, manually delineated, from one subject in the Natural Scenes Dataset [3]. A linear model using the fine-tuned model’s features achieves a higher encoding performance for fMRI responses than untuned AlexNet in all of the 9 subjects tested, with an average improvement of the average correlation of 8.1%. This indicates that the ROI-specific CL fine-tuned models extract generalizable image features that are more predictive of the brain response in early visual areas than models solely trained for image classification.
- After CL-based ROI-specific fine-tuning, we examine the CNNs’ predictions and accuracy on three image classification tasks (ImageNet ILSVRC2012 [13], Caltech256 [14], and Places365 [15]) with freshly trained classification heads, and compare to classification heads on top of the untuned (pretrained, but not fine-tuned) feature extraction model. While the CL-tuned models always have lower classification accuracy, the lower accuracies obtained meaningfully vary by ROI, with larger decreases for early ROIs. Additionally, the regression-tuned models have significantly larger drops in classification accuracy.
- We visualize landscapes of the untuned and ROI-specific feature extraction models by performing dimensionality reduction on the Bhattacharyya dissimilarity created from pairwise comparisons of the downstream task predictions [8]. We note meaningful arrangement of early versus later visual ROIs and divergence between the early visual ROIs in the left and right hemispheres. Similar landscapes can be obtained from representational similarity analysis (RSA) [16] of the task predictions or the underlying features. On test images, we use RSA to confirm that the CL-tuned representations are more similar to the ROI’s neural response than the untuned AlexNet representations.
- We highlight the potential for in-silico experimentation with ROI-specific fine-tuned models by examining patterns in the images generated by Stable Diffusion [17] when using the CL-tuned models for different ROIs and subjects to prompt it. We find meaningful variations between an early (ventral V1) and higher visual-processing ROI (EBA) that are consistent across the 8 subjects. Quantitative assessment of the feature representations of the generated images show that they are similar to the original representations but that the generative process loses some information and lowers the estimated intrinsic dimensionality [18].

## Related work

### Linear encoding models for fMRI

Encoding models for the visual cortex that attempt to predict the neural response to an image often rely on two-stage modeling [19]. First, a predetermined nonlinear transformation is used to extract relevant features from stimulus images. Second, for each voxel, a linear model is trained to predict the voxel response as a linear function of the image features. Typically, ordinary least squares or ridge regression [20] ($\ell_{2}$-regularized mean-squared error minimization) are used as the feature covariance matrix inversion is shared across all voxels. The nonlinear feature mapping may vary based on what ROI the voxel is in, and can be informed by the hypothesized role of the ROI in question. The work by [6] used a receptive-field model based on Gabor wavelets to predict activity of voxels in early visual regions, namely V1, V2, and V3. Semantic labels that describe basic object and action categories for an image have been used as features for encoding ROIs higher in the visual processing stream [7]. Along similar lines, the work by [21] compared linear encoding models using Fourier power characteristics, subjective distance, and object categories as different possible feature representations and found that while the Fourier characteristics were more predictive of voxels in V1, the object categories were more predictive of voxels in ROIs higher in the visual processing stream like RSC, OPA, and PPA (an ROI associated with encoding information about the spatial content of scenes [22]). Multiple feature spaces can be used together through group ridge regression [19,23].

### Neural network-based encoding models

More recently, representations from convolutional neural networks (CNNs) have been used as the basis for linear encoding models [24,25]. The work by [24] extracted the activations from one layer of a CNN, applied spatial subsampling for dimensionality reduction, then fit a ridge regression model using the features to predict the activations of each voxel. The work by [25] used a similar approach but without the intermediate dimensionality reduction. The works by [24] and [25] showed that the hierarchy of layers of CNNs trained on object recognition tasks had correspondences with and could be used to predict voxels throughout the human visual processing hierarchy. Specifically, they showed that the early convolutional layers of CNNs tended to be more predictive of voxels in early-visual regions, and that the later layers in CNNs tended to be more predictive of ROIs higher in the visual processing stream.

Recently, more modern approaches that use various neural network architectures have been proposed for fMRI encoding in the visual cortex. These approaches allow for more flexibility in the learned feature representations of stimulus images. The work by [26] used the pretrained AlexNet as the feature extraction model for predicting voxel activations in early visual regions. The work by [27] trained a variational auto-encoder that can be used for both encoding brain responses and decoding viewed images, although they found lower encoding accuracies with this approach when compared to a CNN model. [28] used different deep neural networks with the same architecture trained for different vision tasks and found correspondence between the task and ROI, for example, edge detection for V1 and semantic tasks for later layers. At the same time as the writing of this paper, the work by [29] showed that neural networks trained from scratch to predict neural activity in V1-V4 using various types of hierarchies can outperform pretrained AlexNet. Our work differs in the choice of CL for fine-tuning, which has not been seen in any previous work on encoding models. However, for the purpose of fMRI decoding, a recent paper used CL to learn embeddings of fMRI responses in the image space of a pretrained CLIP model that could be used for decoding and reconstruction of the visual stimuli [1].

### Contrastive learning

Contrastive learning (CL) [30] is a framework that has been adopted for self-supervised learning [31,32] and mutual information estimation [33,34]. The goal is for non-linear embeddings from one [4] or more [5] input spaces, based on knowledge of positive pairings of input examples that share information,should be more similar after embedding than negative pairings of independently sampled examples. The positive and negative pairs may be constructed in various ways. In the SimCLR [4] formulation, CL is used for self-supervised learning on image datasets without labels. Positive pairs are created by associating two modified views of the same image, created by data augmentations like rotation, cropping, and color distortion. The augmentations are chosen such that they clearly encode the same information for downstream image classification tasks. The goal is to learn an embedding that ignores the variance due to augmentation and preserves the shared information between views. CL creates representations useful for downstream tasks [35], which has been theoretically justified in the recent work by [12]. In our case, the two views stem from the same image, but consist of the original stimulus images and the corresponding brain response. Therefore, a positive pair in our case is an image and its corresponding neural response (beta coefficients extracted from the fMRI BOLD response), and a negative pair is an image and a fMRI response for some other non-matching image. This is akin to pairing of an image with a caption, as in the CLIP model [5], which has been previously used for neural decoding of images from fMRI [36].

Several previous works have assessed the ability of neural networks which were trained using self-supervised methods such as CL to predict neural responses in the visual cortex. Prior work [37,38] found that linear encoding models that used image representations from ResNets [39] trained using CL achieved better encoding performance than encoding model that used features from models trained with supervised techniques, even across networks with similar architectures [40]. The key novelty of our approach is that we use CL to fine-tune a neural network using the neural responses themselves. Furthermore, we conduct analyses of how the CL fine-tuning affects the best intermediate feature layer for encoding, the image representations of the model, performance on downstream tasks, and the use of the model representations as prompts for a generative task.

## Methodology

### Dataset

The dataset used for the modeling and majority of our analysis is the Natural Scenes Dataset [3], which consists of natural image stimuli and the average beta coefficients across multiple trials of a generalized linear model for the BOLD response captured by high-resolution 7T fMRI. Each of the eight subjects viewed approximately 9,000–10,000 distinct images across 3 trials (approximately 30,000 trials in total across multiple sessions). While beta coefficients were fit using voxel-specific hemodynamic response functions to each trial, our analysis uses the average across the 3 trials as provided by [3]. The images used as stimuli are from the Common Objects in Context (COCO) database [41]. Data is available for voxels in the visual cortex that were responsive to the visual stimuli, with approximately 20,000 total voxels per subject across the left and right hemispheres. A wide array of ROIs are available, which were manually segmented using functional properties. Most ROIs have both a left hemisphere (LH) and right hemisphere (RH) group of voxels, and we treat each hemispheric region as its own ROI. The data was organized as part of the Algonauts Challenge, an open competition where participants attempted to predict visual cortex responses to complex natural images [42]. We split the dataset (image and average response pairs) for each subject such that 85% of the data is used for training and validation of the neural network fine-tuning, and the remaining 15% is used for fitting and evaluating the encoding models, with the split consistent across all ROIs for a given subject (but not across subjects, since different subjects viewed different images during the trials). The size of the test sets for subjects 1–8 are 1477, 1477, 1363, 1317, 1477, 1363, 1477, and 1317 images, respectively.

### Encoding model overview

In our proposed approach, for each subject and ROI we fine-tune the parameters of an instance of a pretrained AlexNet CNN to act as the feature extraction model. This results in a total of 280 feature extraction models, where each model is specific to a single subject and hemisphere-specific ROI.

For both our proposed approach and baselines we fit neural encoding models for each subject’s ROIs using three stages: first, feature extraction is performed by passing the stimulus image to the pretrained or fine-tuned CNN, and the activations at a particular layer are treated as features; second, because early CNN layers have high-dimensional activations, the number of dimensions is reduced via principal component analysis (PCA); and third, a linear model is fit from these features to each voxel using ridge regression ($\ell_{2}$-normalized linear regression).

To quantify the accuracy of the prediction, we evaluate the average correlation between the predicted responses and the true responses and average across all voxels in the ROI, as described in Accuracy Metric.

### Baseline feature extraction models

As a baseline feature extraction model we consider the AlexNet CNN [9] pretrained on ImageNet [43]. All encoding models are specific to a particular subject- and hemisphere-specific ROI, but the base model (with or without fine-tuning) serves as a backbone for all image feature extraction models.

To match the expected input for AlexNet, the stimulus images are all resized to 224-by-224 pixels and their means and standard deviations are normalized so that the means across the 3 colors channels are 0.485, 0.456, and 0.406, and the standard deviations across the color channels are 0.229, 0.224, and 0.225 (a standard preprocessing step for using an AlexNet model that was pretrained on ImageNet).

#### Pretrained AlexNet model.

We consider using the different layers of AlexNet (either after one of the five convolutional layers or after one of the 3 fully connected layers), the dimensions of which are listed in Table 1, for feature extraction.

**Table 1:** Dimensions of the output at 8 different AlexNet layers, where the spatially organized convolutional layers (conv) have been vectorized. The final layers are fully connected (FC), with the final output being 1000 dimensions.

Layer	Conv-1	Conv-2	Conv-3	Conv-4	Conv-5	FC-1	FC-2	FC-3
**Dimension**	46,656	32,448	64,896	43,264	9216	4096	4096	1000

The pretrained AlexNet model acts as the starting point for all functions performing image feature extraction. Tapping into the AlexNet model at layer l∈{1,...,L} with *L* = 8, we can select different amounts of processing of the original image for different ROIs.

Before the linear model is fit, for each layer l∈{1,...,L}, we apply PCA to reduce the dimensionality of the representation to be constant for all layers, taking the first *d* = 1000 principal components of the training images’ representations for a subject (as training images differ by subject).

For each ROI, we select the best representation layer for linear encoding from the pretrained AlexNet model by evaluating the cross-validated performance of an $\ell_{2}$-regularized linear model fit for each voxel in the ROI using the dimension-reduced activations by evaluating the eight possible layers l∈{1,...8}. After fine-tuning, we do the same search with the fine-tuned model to find the optimal layer for encoding with the tuned models.

### Mathematical formulation of neural encoding framework

Let x∈ℐ represent a single image (for AlexNet, $ℐ\subset {\mathbb{R}}^{3\times 224\times 224}$). For a given subject and ROI, let $𝐲\in {\mathbb{R}}^{v}$ represent the corresponding *v*-dimensional cortical response in the ROI. The encoding model is a function $Enc:ℐ\to {\mathbb{R}}^{v}$ that predicts the *v*-dimensional cortical response as a composition of two models: the ROI-specific feature extraction model $f:ℐ\to {\mathbb{R}}^{d}$, where *d* is the output dimension of the feature extraction model, and a linear model described by coefficients and biases $𝐁\in {\mathbb{R}}^{v\times (d+1)}$. Specifically, let $\tilde{𝐚}=[𝐚,1{]}^{⊤}$ denote the activations at the output of *f* appended with a 1, then $\hat{𝐲}=𝐁\tilde{𝐚}$ is the prediction of the cortical response in the ROI to image *x*.

Let ℱ denote the family of feature extraction models. Ideally, the goal is to find the feature extraction model for the ROI that minimizes the expected sum of mean-squared error across voxels $\|𝐲-\hat{𝐲}{\|}_{2}^{2}=\sum_{k=1}^{v}(y_{k}-{\hat{y}}_{k}{)}^{2}$, giving an optimal regression for each voxel. This is stated as the following optimization problem:


$$\begin{array}{c} \min_{f\in ℱ}\min_{𝐁\in {\mathbb{R}}^{v\times (d+1)}}{𝔼}_{\left(x,𝐲\right)}[\|𝐲-𝐁[f(x),1{]}^{⊤}{\|}_{2}^{2}]. \end{array}$$ (1)

In practice, limited training data $\{(x_{i},{𝐲}_{i}){\}}_{i=1}^{n_{train}}\subset ℐ\times {\mathbb{R}}^{v}$ is available to compute the expectation, and the capacity of ℱ, especially when it is a deep CNN, leads to overfitting.

To improve generalization, we use a three stage approach. First, we select a particular layer l∈{1,...,L} of a CNN after the CNN has been fine-tuned with a contrastive learning loss function to serve as the feature extraction model; second, we apply principal component analysis (PCA) to reduce the dimension of the features; and third, we apply $\ell_{2}$-regularized linear regression to the reduced feature set to form the linear model. The PCA and regularization both reduce the effect of overfitting, but the PCA step is also essential to reduce the computational demands of forming and inverting a high-dimensional covariance matrix.

In the first stage of our proposed approach (and the regression-tuned baseline), we fine-tune an instance of the pretrained AlexNet model using contrastive learning for each ROI and individual. Next, given an ROI-tuned feature extraction model consisting of the fine-tuned AlexNet at the *l*th layer with output dimension $d_{l}$, denoted $f_{l}:ℐ\to {\mathbb{R}}^{d_{l}}$, we apply PCA to reduce the dimension, yielding the dimension-reduced activation vector ${𝐚}_{i}=f(x_{i})={𝐔}^{⊤}f_{l}(x_{i})$, where $𝐔\in {\mathbb{R}}^{d_{l}\times k},k\leq d_{l}$, is the matrix of the leading *k* eigenvectors defining the principal components of the activations in the training set $\{f_{l}(x_{i}){\}}_{i=1}^{n_{train}}$. Given ${\tilde{𝐚}}_{i}=[{𝐚}_{i},1]\in {\mathbb{R}}^{k+1}$, an $\ell_{2}$-regularized linear model is fit with voxel-specific penalty terms $\alpha^{1},...,\alpha^{v}$ (selected by cross validation) as


$$\begin{array}{c} \min_{𝐁\in {\mathbb{R}}^{v\times k+1}}\sum_{i=1}^{n_{train}}\|{𝐲}_{i}-𝐁{\tilde{a}}_{i}{\|}_{2}^{2}+\sum_{j=1}^{v}\alpha^{j}\sum_{i=1}^{k}B_{j,i}^{2}. \end{array}$$ (2)

**Fig 1:**
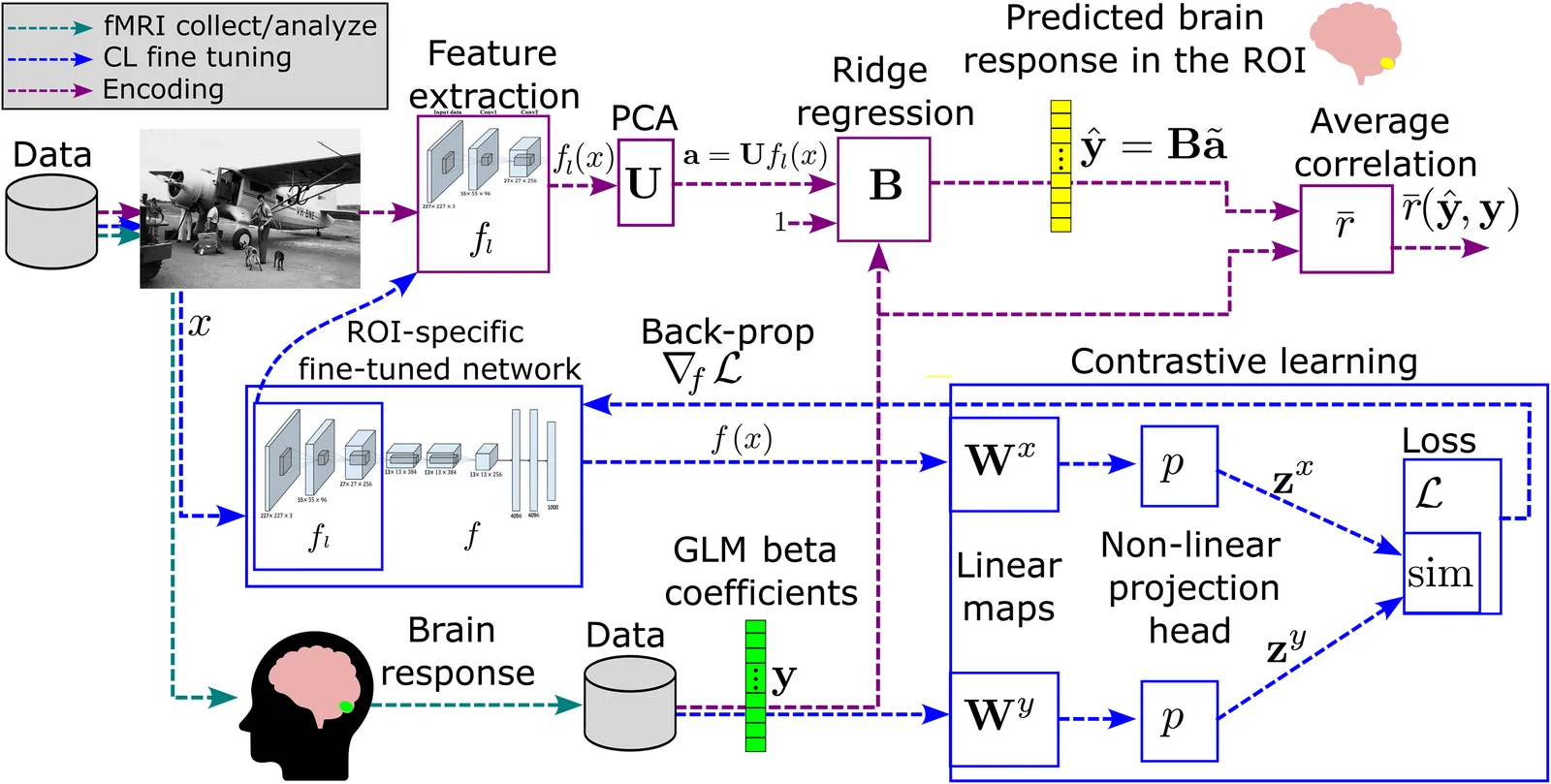
Overview of the proposed approach. (Green arrows) The brain response to natural images (GLM beta coefficients fit to the fMRI) are organized into visual ROIs. (Blue arrows) Contrastive learning (CL) is used to fine-tune the AlexNet CNN *f* based on a loss ℒ that maximizes the cosine similarity sim of processed image-response pairs ${𝐳}^{x},{𝐳}^{y}$, contrasted to random pairs ${𝐳}^{x^{\prime }},{𝐳}^{y}$ with $x^{\prime }\neq x$ (not depicted in the figure). $z^{x}$ is obtained by passing the image *x* through the AlexNet CNN *f* and then a linear projection ${𝐖}^{x}$ and the shared non-linear projection head ***p*.** $z^{y}$ is obtained by passing the fMRI response 𝐲 through a linear projection ${𝐖}^{y}$ and then the shared non-linear projection head *p*. *f* is updated by back-propagation using the gradient $\nabla_{f}ℒ$. (Purple arrows) An encoding model for the ROI uses the output from layer *l* of the fine-tuned AlexNet, applies PCA, and then fits a $\ell_{2}$ penalized linear model via ridge regression. Performance of the encoding predictions are assessed on held-out images via the correlation coefficient $\bar{r}$ averaged across an ROI’s voxels.

The predicted neural response to image $x_{i}$ is then $\hat{𝐲}=𝐁{\tilde{a}}_{i}$. The overview of our approach is depicted in Fig 1.

### Accuracy metric

To assess the performance of a model, we compute the average correlation coefficient between the predicted response and the true response across all voxels in that ROI


$$\begin{array}{c} \bar{r}(\hat{𝐲},𝐲)=\frac{1}{v}\sum_{j=1}^{v}r({\hat{y}}_{j},y_{j}), \end{array}$$ (3)

where $r({\hat{y}}_{j},y_{j})$ is the Pearson correlation coefficient between ${\hat{y}}_{j}$ (the predicted responses for the *j*th voxel of the ROI) and the true responses $y_{j}$. Accuracies over the entire test set are computed as the average $\bar{r}(\hat{𝐲},𝐲)$ over all test set images.

#### Encoding model training, hyper-parameter selection, and evaluation.

For encoder model training, hyper-parameter selection (ROI-specific layer of the AlexNet model for feature extraction and voxel-specific regularization penalty), and evaluation we use the 15% of the original dataset that was held out from the feature extraction model training. We perform 5-fold cross validation over this 15%. For each of the 5 folds, linear encoding models for each voxel (and hyper-parameter combination) are trained on the remaining 4 folds, then these models are then tested on the held out fold.

For each ROI, a single AlexNet layer is selected that has the best average performance (average correlation on test) across the folds and voxels using each voxel’s penalty term $\alpha^{j}$ for the regularized linear regression selected from the log-spaced grid $\left[10^{-1},10^{0},...,10^{7}\right]$ that works best averaged across folds given that layer. It should be noted that the corresponding average test correlation for a layer is also the final encoding accuracy for that ROI (so that the final test correlation for an ROI is computed with all the voxels using the same output layer, but voxel-specific penalty terms that are fixed across folds). Note that while the average cross-validation accuracy is used to select the layer and penalty, the encoding models are never trained on test data. As this CV hyper-parameter selection is applied uniformly to all feature extraction models (untuned, CL-tuned, and regression-tuned), it is a fair evaluation.

#### Neural network regression model.

Based on Eq 1, and to parallel the CL-approach, we create a fine-tuning baseline that tries to predict the voxel activity in a given subject and ROI from the final AlexNet activations of dimension *d* = 1000. The feature extraction model (and subsequent layers) is optimized using the mean squared error between the predicted and actual fMRI response as the loss function,


$$\begin{array}{c} f=\underset{f\in ℱ}{\text{arg min}}\min_{{𝐁}_{O}\in {\mathbb{R}}^{v\times (d+1)}}\sum_{i=1}^{n_{train}}\|{𝐲}_{i}-{𝐁}_{O}[f(x_{i}),1{]}^{⊤}{\|}_{2}^{2}, \end{array}$$ (4)

where the parameters of both the linear mapping at the output and the entire AlexNet are optimized during training, with the latter initialized as the pretrained AlexNet CNN. After fine-tuning, the activations at a specific layer is dimension-reduced via PCA and an encoding model is fit to predict voxel responses. While indirect, i.e., the encoding model built at the output of the fine-tuned CNN is not actually used, using regression to fine-tune the image feature extraction model specific to a subject and ROI has the potential to outperform the baseline pretrained model, but not overfit as much as a direct encoding model as in Eq 1. The hyper-parameters of the regression-tuned encoding model, layer and voxel-wise penalty are chosen using the same cross-validation approach as in Encoding Model Training, Hyper-Parameter Selection, and Evaluation.

### Contrastive learning for ROI-specific feature extraction

The crucial technique in our proposed approach is the use of a contrastive loss function to fine-tune the ROI-specific feature extraction model. The goal is to learn representations of images that emphasize features that are relevant for a given ROI. Contrastive estimation uses machine learning to estimate log density functions [30] based on discriminating ‘positive’ true data from ‘negative’ data created from noise or a source of randomness. In the context of paired random variables, the noise is independent observations, and estimates of the log-density of the conditional distribution have been shown to lower-bound mutual information [33]. Maximizing this lower-bound provides an objective for optimizing the image feature extraction model.

Inspired by the SimCLR framework [4], which optimizes a feature extraction network by using differently augmented pairs of the same original image as positive pairs and augmented pairs of different images as negative pairs, we propose a multimodal CL approach akin to the CLIP model [5] that takes a given image $x_{i}\in ℐ$ paired with its corresponding fMRI response ${𝐲}_{i}\in 𝒴$ as a positive pair, and an image $x_{i}$ and a disparate fMRI response ${𝐲}_{j}$, i≠j, as a negative pair. The contrastive loss aligns 𝐲, the response in the ROI, to the output of *f*. After fine-tuning, the feature extraction model *f* uses the features extracted at layer *l* of *f*.

Given a batch of *K* paired vectors in the same space $\{({𝐳}_{i}^{x},{𝐳}_{i}^{y}){\}}_{i=1}^{K}$, a contrastive loss inspired by SimCLR [4] is


$$\begin{array}{c} ℒ(\{({𝐳}_{i}^{x},{𝐳}_{i}^{y}){\}}_{i=1}^{K})=\frac{1}{K}\sum_{i=1}^{K}-\log\left(\frac{\exp(\frac{1}{\tau }sim({𝐳}_{i}^{x},{𝐳}_{i}^{y}))}{\sum_{j\neq i}\exp(\frac{1}{\tau }sim({𝐳}_{i}^{x},{𝐳}_{j}^{y}))}\right), \end{array}$$ (5)

where $sim(𝐳,{𝐳}^{\prime })=\frac{\langle 𝐳,{𝐳}^{\prime }\rangle }{\|𝐳{\|}_{2}\|{𝐳}^{\prime }{\|}_{2}}$ is the cosine similarity and τ>0 is a temperature parameter. While slightly different due to the dropping of the positive pair j≠i in the denominator, this contrastive learning loss function can be related to a lower bound on mutual information [33], as discussed in S1 Appendix.

To map the ROI-specific image features and the ROI response to a common space where we can evaluate cosine similarity, we propose to use two linear mappings denoted by matrices ${𝐖}^{x}$ and ${𝐖}^{y}$ to map the fine-tuned activations 𝐚=f(x) and 𝐲, respectively, to a shared vector space ${\mathbb{R}}^{d_{h}}$. As in the SimCLR architecture [4], this is followed by a shared non-linear function, specifically, a multi-layer ‘perceptron’ (MLP), referred to as a projection head $p:{\mathbb{R}}^{d_{h}}\to {\mathbb{R}}^{d_{z}}$ (we note that CLIP [5] does not use a projection head). The vectors in the shared space are then ${𝐳}^{x}=p({𝐖}^{x}f(x))$ and ${𝐳}^{y}=p({𝐖}^{y}𝐲)$. The model architecture is summarized by Fig 1.

The optimization problem is


$$\begin{array}{c} f,{𝐖}^{x},{𝐖}^{y},p=\underset{f\in ℱ,{𝐖}^{x},{𝐖}^{y},h}{\text{arg min}}{𝔼}_{\pi }\left[ℒ(\{(h({𝐖}^{x}f(x_{\pi (i)})),h({𝐖}^{y}{𝐲}_{\pi (i)})){\}}_{i=1}^{K})\right], \end{array}$$ (6)

where the expectation is over mini-batches drawn from the training set defined by the mapping function $\pi :\{1,...,K\}\to \{1,...,n_{train}\}$ created by sampling *K* integers from $\left\{1,...,n_{train}\right\}$ without replacement. Based on the bound on mutual information (see S1 Appendix), *K* should be as large as possible [4,33], but is limited in practice since the computation of ℒ has quadratic complexity with respect to the batch size.

Once the subject and ROI-specific model is fine-tuned, the same layer and penalty term cross-validation search as in Mathematical Formulation of Neural Encoding Framework is applied to build the final encoding model. Essentially the subsequent layers, which are also fine-tuned, are discarded for the purposes of encoding, as in the regression-tuned baseline. Dropping later fine-tuned layers has precedence in self-supervised learning [44]. As in the other baselines, the activations at this layer are projected onto the first *k* = 1000 principal components based on the training set and $\ell_{2}$-regularized linear regression is used to fit the encoding model.

### Implementation details

We set the dimensionality of the vector spaces based on *v*, the number of voxels in the ROI, with $d_{h}=\lfloor 0.8v\rfloor$, $d_{z}=\lfloor 0.2v\rfloor$, and the temperature as τ=0.3. In comparison, for SimCLR [4], τ∈{0.1,0.5,1.0}, while for CLIP [5], τ is initialized to 0.07 and limited to 100 but updated in terms of the parameter t=log(τ). The projection head consists of three fully connected layers, with the first two layers each being followed by a batch normalization layer and then a ReLU activation function. The first two layers of the MLP projection head keep the dimension of the projection *h* the same, and the final layer reduces the dimension to $d_{z}$.

We use Scikit-Learn to fit the regularized linear models [45] and PyTorch with CUDA to fine-tune the neural networks [46]. For all subjects and ROIs, the fine-tuning optimization uses batch sizes of *K* = 1024, 30 epochs for CL and 75 epochs for regression, and an Adam optimizer with a learning rate of $2.5\cdot 10^{-5}$ for contrastive learning and 10^-4^ for regression, betas of 0.9 and 0.999, an epsilon of 10^-8^, and no weight decay. The number of training epochs for each method was selected by using a subset of 30 randomly selected ROIs from across the 8 subjects, splitting up the training data further into 85% training and 15% validation, and manually noting when the validation loss had converged for all the ROIs. Learning rates were selected based on the validation loss for a few ROIs, but the grid search was not extensive. We fine-tune the models using T4, V100, and P100 GPUs, on which the training takes roughly 2 minutes per epoch.

### Pooled models

For each ROI, we also train a pooled model using the data from all subjects which have that ROI to fine-tune a single shared AlexNet backbone. The architecture is identical to the one described for subject-specific models in Contrastive Learning for ROI-specific Feature Extraction, but with a separate linear mapping ${𝐖}_{s}^{y}$ for each ROI and subject *s*, which is required because of the varying voxel mappings and dimensionality for the same ROI across different subjects. To assess the impact of the dimensionality of the latent space $d_{h}$, for each ROI we train one pooled model with $d_{h}$ equal to the average voxel dimension of all subjects with that ROI (rounded to an integer), and another pooled model with $d_{h}$ equal to the maximum voxel dimension over all ROIs in all subjects. The same training methodology and optimization hyper-parameters as in Implementation Details are used. Notably, one epoch of training involves a pass over the training images and corresponding fMRI responses for each subject, rather than just one subject, which increases the training set size. The performance of the pooled models gives an indication of how transferable the features learned from fine-tuning on one subject are to other subjects.

### External dataset validation

A valid concern is whether the CL-tuned models are transferable beyond the Natural Scenes Dataset with its high-resolution fMRI and its ROI definitions. In order to investigate whether the CL-tuned models are extracting images features that are predictive of the fMRI response in corresponding ROIs, we validate them for brain encoding on the Natural Object Dataset (NOD) [11], which uses the Human Connectome Project Multi-Modal Parcellation (HCP-MMP) for ROI definitions [47]. Notably, NOD uses a 3T scanner and also uses stimulus images drawn from ImageNet for some subjects. For our validation, we use the responses to the ImageNet images for subjects 1–9, each of which have recorded responses to 4000 unique ImageNet images (whereas the remaining 21 subjects in NOD only have responses to 1000 images each). Because the fMRI responses are relatively noisy, we follow the encoding experiments in [11] and use only the 50 voxels in each ROI with the highest noise ceiling estimates, which were estimated in [11] as the 50 voxels which were best predicted by a population receptive field model fit to retinotopic mapping data.

We evaluate on the early visual ROIs V1-V4 from the ventral stream in both left and right hemisphere for each subject. For each ROI and subject, we use the CL-tuned pooled model for the corresponding ROI from NSD as the feature extraction model, and following Encoding Model Training, Hyper-Parameter Selection, and Evaluation, we evaluate the 5-fold cross-validated performance of an $\ell_{2}$-regularized linear encoding model fit for each voxel in the ROI using the dimension-reduced activations from each of the 8 possible output layers from the CL-tuned AlexNet model, along with untuned AlexNet as a baseline. Note that this cross-validation technique is the same as the one used to evaluate the encoding models for NSD, but here we use the entire NOD dataset since none of the data was used for AlexNet fine-tuning.

### Downstream classification tasks

To quantitatively analyze the CL-tuned feature extraction models, we build classifiers on top of the untuned and ROI fine-tuned models for standard image classification tasks. We use three different image classification datasets: ImageNet ILSVRC2012 [13], which is the dataset used in the AlexNet pre-training, Caltech256 [14], and Places365 [15]. ILSVRC2012 contains 1000 different classes, Caltech256 has 255 object classes (plus 1 catch-all “clutter” class), and Places365 has 365 classes. The Caltech256 and Places365 datasets provide external validation of the fine-tuned models, with the former containing classes focusing on specific objects or figures like bears, helicopters, and microwaves, while the latter’s categories are more general scenes like bedrooms, cafeterias, and staircases. We use the validation split of ILSVRC2012 containing 50,000 images, the entire Caltech256 dataset containing 30,607 images, and the validation portion of Places365, which has 36,500 images. For each, we randomly split the dataset into 85% for training and 15% for testing. We use the penultimate layer’s 4096-dimensional activations of the tuned or pretrained AlexNet CNN as feature representations of the images, fit a multinomial logistic regression model to predict the labels of the training data, and then evaluate the accuracy of the classifier in predicting the labels of the testing images. For ILSVRC2012, this allows us to probe how much fine-tuning (CL or regression) degrades the downstream performance as the model focuses on features relevant for predicting the activity in a subject’s ROI, possibly forgetting features useful for the classification task. For the Caltech256 and Places365 tasks, this accuracy indicates how well the features of the untuned and fine-tuned models can be used for datasets outside of the one that they were initially trained on. In summary, we train a classifier on top of the pretrained AlexNet CNN (replacing its own classification head), each subject-ROI-specific CL fined-tuned model, each pooled-subject, ROI-specific CL fine-tuned model (with both choices of embedding layer dimension), and each subject-ROI-specific regression-tuned model.

### Model landscapes

Given a set of classification models, prior work [8] proposes an approach for visualizing the principal components of their pairwise dissimilarities based on the average similarity of their predictions across a test set. For *M* models, the pairwise dissimilarity matrix $𝐃\in [0,\infty {)}^{M\times M}$ is computed as the average of the negative logarithm of the Bhattacharyya similarity B of the class prediction probabilities across a test set of size $n_{test}$, $D_{m,m^{\prime }}=\frac{1}{n_{test}}\sum_{i=1}^{n_{test}}-\log(B({𝐩}^{m}(x_{i}),{𝐩}^{m^{\prime }}(x_{i}))$, $m,m^{\prime }\in \{1,...,M\}$, where $B(𝐩,𝐪)=\sum_{k=1}^{C}\sqrt{p_{k}q_{k}}$ and ${𝐩}^{m}(x_{i})$ represents the *m*th classification model’s probabilities estimates across the *C* classes for image $x_{i}$. Multidimensional scaling (MDS) is applied to derive a 2D embedding for the *m*th model with coordinates $\left[U_{m1}\sqrt{\left|\Lambda_{11}\right|},U_{m2}\sqrt{\left|\Lambda_{22}\right|}\right]$, where $U_{mp}$ is the *m*th entry of the eigenvector corresponding to the *p*th largest magnitude eigenvalue $\Lambda_{pp}$ of the matrix $-\frac{1}{2}𝐇𝐃𝐇=𝐔\Lambda {𝐔}^{⊤}\in {\mathbb{R}}^{M\times M}$, where $𝐇={𝐈}_{M\times M}-\frac{1}{M}𝐉$ is the centering matrix and 𝐉 is a matrix of all ones. Beyond visualization, the correspondence of the 2D embeddings to ROI groupings can be quantified by the well-known Silhouette index [48]. We estimate jackknife confidence intervals (95%) around the Silhouette index by calculating Silhouette indices after leaving out each subject in turn (we recompute MDS in this loop).

We also apply representational similarity analysis (RSA) to compare representations (e.g., model embeddings or the brain response in an ROI) [16]. In this case, $D_{m,m^{\prime }}=1-\rho ({𝐝}_{m,𝔡},{𝐝}_{m^{\prime },{𝔡}^{\prime }})$ where the Spearman rank correlation ρ is computed between the entries of the representation similarity matrices ${𝐝}_{m,𝔡}=[𝔡(r_{m}(x_{i}),r_{m}(x_{j})){]}_{1\leq i<j\leq n_{test}}$ using the representation $r_{m}:ℐ\to {\mathbb{R}}^{d_{m}}$, and symmetric dissimilarity measure $𝔡:{\mathbb{R}}^{d_{m}}\times {\mathbb{R}}^{d_{m}}\to \mathbb{R}$. RSA is a general tool that can measure how similar the representation (brain or model) are in terms of the pairwise ordering of the $n_{test}$ choose 2 dissimilarities. Here, we adopt either the Euclidean distance $𝔡(𝐚,{𝐚}^{\prime })=\|𝐚-{𝐚}^{\prime }{\|}_{2}$ or the ‘correlation distance’ dissimilarity $𝔡(𝐚,{𝐚}^{\prime })=1-r(𝐚,{𝐚}^{\prime })$, for two representations $𝐚,{𝐚}^{\prime }\in {\mathbb{R}}^{d_{m}}$.

### Generating stable diffusion images from CL-tuned models

As a simple example of the type of “in-silico” experiments that can be conducted with the brain-tuned models for different ROIs, we use the CL-tuned models to generate prompts for Stable Diffusion [17] to examine perceptual differences in the generated images from the models tuned on different ROIs. Stable diffusion models often use a CLIP text embedding as the prompt to handle image generation. Image-to-prompt [49] proposes a method to directly use an image embedding as the prompt. However, it requires that the image and text embeddings lie in the same learned latent space. Since the training of the stable diffusion model [17] is conditioned on CLIP ViT-L/14, it is necessary to align the image embedding spaces first. We formulate the space alignment problem as the well-known orthogonal Procrustes problem [50], which aims to find a matrix with orthonormal columns that minimizes the mean-squared error as a regression problem between the embeddings. It has a closed-form solution in terms of the singular value decomposition of the product of the two embedding matrices. For each CL-tuned model that is specific to a subject and ROI, we use the *d* = 1000-dimensional embeddings of the fine-tuned model *f* for the NSD training images and the corresponding embeddings produced by CLIP ViT-L/14. Note that while CLIP embeddings of images capture semantics, they may not capture information about spatial arrangement.

## Results

At the coarsest level, we divide the ROIs into two coherent groups [42]: early visual regions (V1, V2, V3, hV4) and higher visual regions (body-, face-, place-, or word-selective regions); the latter contains EBA, OFA, OPA, OWFA, and others. Thus, ROIs are characterized according to 5 main groups: early visual regions, body-selective regions, face-selective regions, place-selective regions, and word-selective regions. The early visual regions are further defined (V1v, V1d, V2v, V2d, V3v, V3d, hV4) based on population receptive field mapping (pRF) experimental results [3]. The body- (EBA, FBA-1, FBA-2, mTL-bodies), face- (OFA, FFA-1, FFA-2, mTL-faces, aTL-faces), place- (OPA, PPA, RSC), and word-selective regions (OWFA, VWFA-1, VWFA-2, mfs-words, mTL-words) were manually created using the results of a functional localizer (fLoc) experiment. Not all ROIs are found on all subjects, as shown in S1 Fig. For more detailed information, see the Natural Scenes Dataset paper [3], especially the supplementary information.

### Pretrained AlexNet model

**Fig 2:**
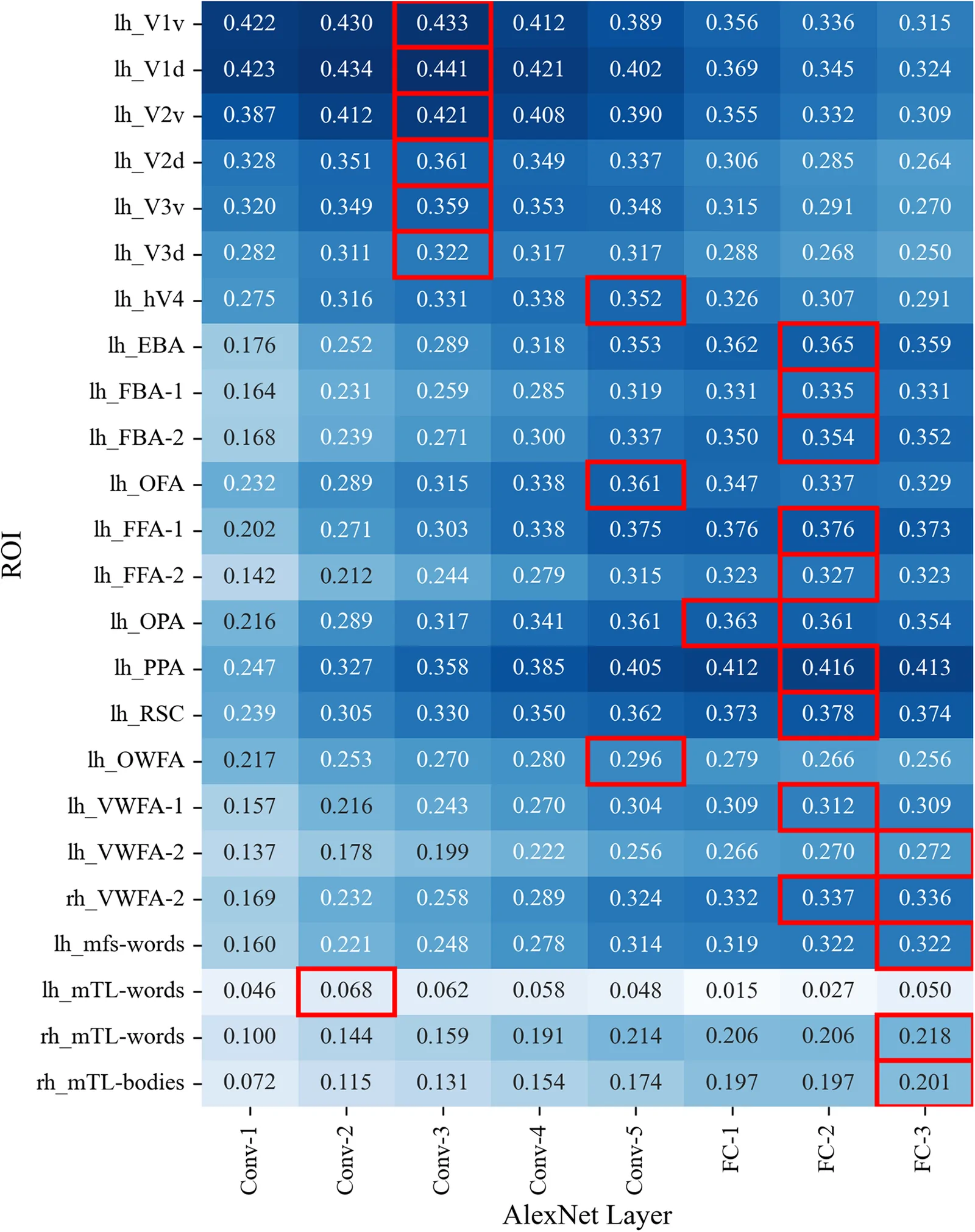
Untuned AlexNet encoding scores per layer. Matrix of encoding scores for each ROI and AlexNet layer, where the entries are the average test encoding accuracy over 5-fold cross validation when a particular AlexNet layer’s activations (after PCA for dimensionality reduction) are used to predict a particular ROI (using the best penalty term for each voxel and layer for ridge regression). Right hemisphere (rh) ROIs are omitted if they have the same best AlexNet layer as their corresponding left hemisphere (lh) ROI.

As described in Baseline Feature Extraction Models, we perform 5-fold cross validation to jointly select the AlexNet output layer and the penalty term for each voxel for the ridge regression for each ROI. We include the layers after each of the 5 convolutional layers and subsequent activation functions and the 3 fully connected layers for a search space of 8 possible output layers. Fig 2 shows the average test cross validation encoding accuracy for each ROI and AlexNet output layer, with the best output layer for each ROI highlighted.

We find that all of the early visual ROIs (V1-hV4) are best predicted by either the second or the third of the five convolutional layers, and that 24 of the 28 higher visual ROIs (counting left- and right- hemisphere splits as different ROIs) are best predicted by the output of one of the two fully-connected layers. Our findings for the most predictive AlexNet layer agree with previous works that suggest that early-visual regions are more similar to the middle convolutional layers (Conv-2 or Conv-3) in CNNs, and ROIs higher in the visual processing stream (PPA, ventral) have higher similarity with the later fully-connected layers [24,25,51].

### Subject-specific models

We first analyze the performance of the subject-specific models described in Contrastive Learning for ROI-specific Feature Extraction on the test set. For each ROI, we evaluate the average Pearson correlation coefficient $\bar{r}(𝐲,\hat{𝐲})$ as described in Accuracy Metric for both baseline models and the CL models, and also judge the performance of the CL models versus the baseline models by computing the percentage of voxels in the ROI that had a higher average test correlation than the baseline model in question across the test images. Subject-specific results for a given subject for each of these groups are calculated as the mean over all ROIs in the group. Table 2 shows the results for the early and higher visual ROI groups.

**Table 2:** Subject-specific encoding performance for early (E) and higher (H) visual ROIs using the untuned AlexNet (Ctrl.), see Baseline Feature Extraction Models; with CL-tuning (CL), see Contrastive Learning for ROI-specific Feature Extraction; and with regression-tuned (Reg.), see Neural Network Regression Model. (Avg. $\bar{r}$) denotes the test set correlation $\bar{r}$, described in Accuracy Metric, averaged across ROIs. The last four columns show the percentage of voxels, averaged across ROIs in the group, for which encoding with the CL-tuned model was more predictive compared to either the untuned model or the regression-tuned model. Using a permutation test, lack of significance at a level of 0.01 is indicated by tilde above.

	Ctrl. Avg. $\bar{r}$		CL Avg. $\bar{r}$		Reg. Avg. $\bar{r}$		CL > Ctrl.		CL > Reg.	
	E	H	E	H	E	H	E	H	E	H
1	0.458	0.383	0.477	0.403	0.498	0.402	97.8%	84.5%	82.0%	49.6%
2	0.446	0.414	0.461	0.436	0.479	0.435	95.1%	88.9%	72.4%	$\tilde{\text{50.4}}$%
3	0.379	0.346	0.385	0.350	0.400	0.357	92.5%	75.5%	74.1%	65.7%
4	0.363	0.335	0.391	0.342	0.391	0.349	94.1%	77.4%	$\tilde{\text{46.6}}$%	66.7%
5	0.419	0.455	0.467	0.474	0.464	0.474	94.6%	91.3%	$\tilde{\text{46.4}}$%	51.5%
6	0.361	0.309	0.385	0.314	0.385	0.315	88.6%	64.0%	$\tilde{\text{49.3}}$%	53.1%
7	0.360	0.328	0.379	0.331	0.388	0.337	91.7%	71.0%	66.6%	62.1%
8	0.304	0.258	0.327	0.262	0.328	0.267	88.8%	68.0%	54.6%	62.5%
	0.386	0.354	0.409	0.364	0.417	0.367	92.9%	77.6%	61.5%	57.7%

Given the limited number of subjects, we only test for improvements of performance at the coarsest ROI grouping (early and higher). We provide other descriptive statistics at the ROI and voxel level, but do not test at these levels. We find that the CL-based fine-tuning produces significantly higher encoding correlation coefficients than the baseline pretrained AlexNet approach in the early visual ROIs (corrected p-value of 0.001 and effect size 0.465 (Cohen’s d) for a paired, one-tailed t-test with Bonferroni correction for 2 tests, sample size of 8 subjects, and significance threshold of α=0.05) as well as in the higher visual ROIs (corrected p-value of 0.009 and effect size of 0.17). While the regression-tuned models have a higher average encoding accuracy than the CL-tuned models in both early visual and higher visual ROIs, the difference is not significant in either the early visual ROIs (corrected p-value of 0.06 and effect size 0.147 (Cohen’s d) for a same test setup as for CL versus untuned), or the higher visual ROIs (corrected p-value of 0.05)).

As an alternative metric, we also examine the average percentage of improved voxels using CL versus the untuned baseline. For the early visual ROIs, the average percentage of improved voxels using CL versus the untuned AlexNet model across subjects is 92.9%, while for the higher visual ROIs, it is 77.6%. Using a permutation test, we compute the distribution of these statistics per subject for the early and higher ROI groups. As the number of voxels per ROI changes per subject, the threshold for significance at a level of 0.01 varies from 51.0–52.5%. Thus, all subjects improvements are significant accounting for Bonferroni correction for two tests (early and higher). As compared to the regression-tuned models, the CL-tuned models improve an average of 61.5% of voxels across subjects for the early visual ROIs and 57.7% of voxels across subjects for the higher visual ROIs. Using the significance thresholds, 5 of the 8 subjects were significantly better for the early visual and 7 of the 8 subjects for the higher visual ROIs. We attribute the relative strength of CL versus regression-tuned in this metric as compared to the mean encoding accuracy to the training objectives of the different approaches. While the regression-tuned models were fine-tuned by minimizing squared error, which heavily penalizes large errors, minimizing the contrastive loss used in CL may be done by predicting a large proportion of voxels well but having higher squared error on a few voxels.

**Fig 3:**
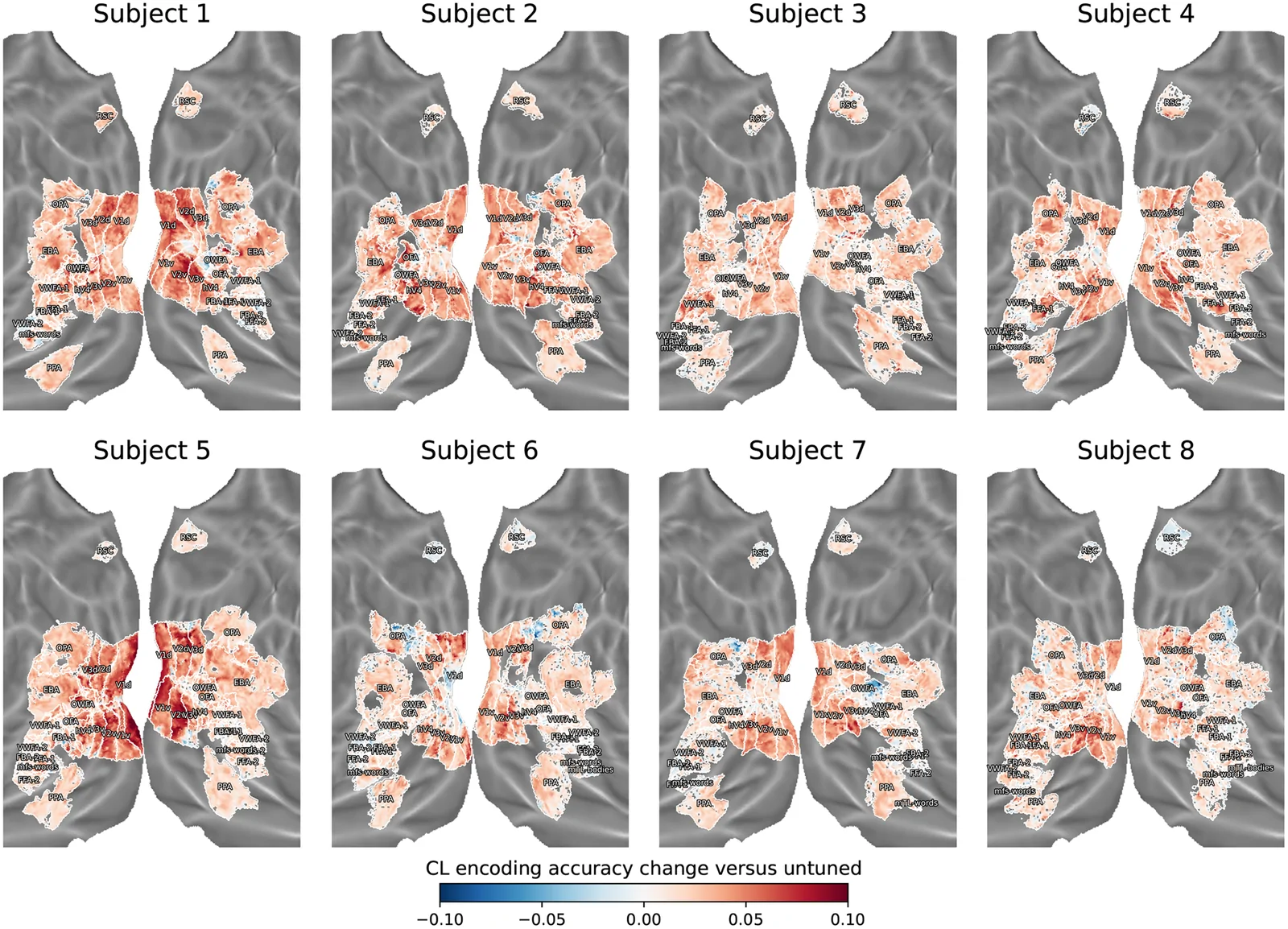
Single-subject CL versus untuned cortical results. Voxel-wise differences in encoding performance *r* visualized on FreeSurfer template for each subject. ‘Hot’ colors indicate voxel improvements with contrastive learning (CL) based fine-tuning versus the pretrained AlexNet.

**Fig 4:**
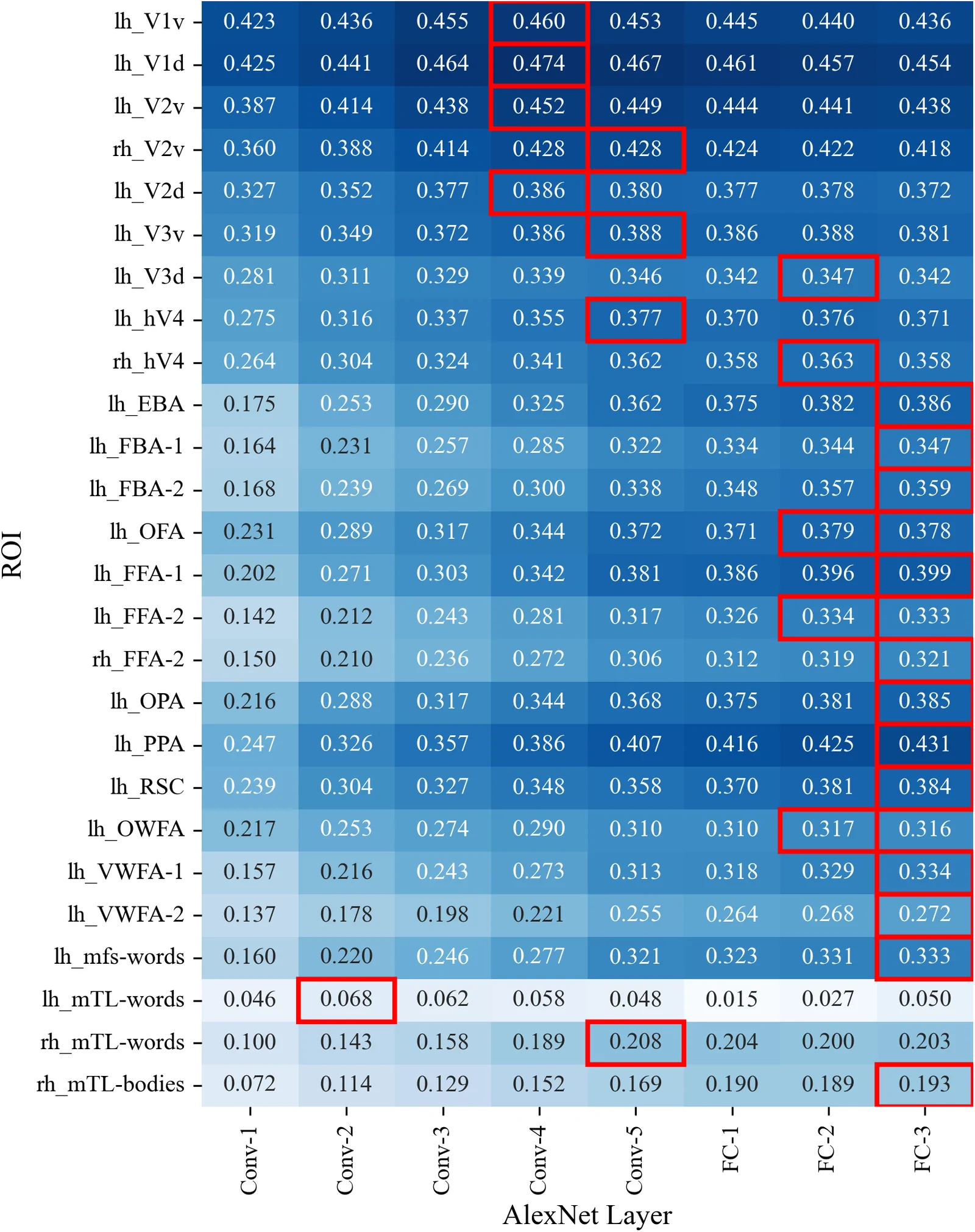
CL-tuned AlexNet encoding scores per layer. Matrix of encoding scores averaged across subjects for each ROI and CL-tuned AlexNet layer, where the entries are the average test encoding accuracy over 5-fold cross validation when a particular AlexNet layer’s activations (after PCA for dimensionality reduction) are used to predict a particular ROI (using the best penalty term for each voxel and layer for ridge regression). Right hemisphere (rh) ROIs are omitted if they have the same best AlexNet layer as their corresponding left hemisphere (lh) ROI.

**Fig 5:**
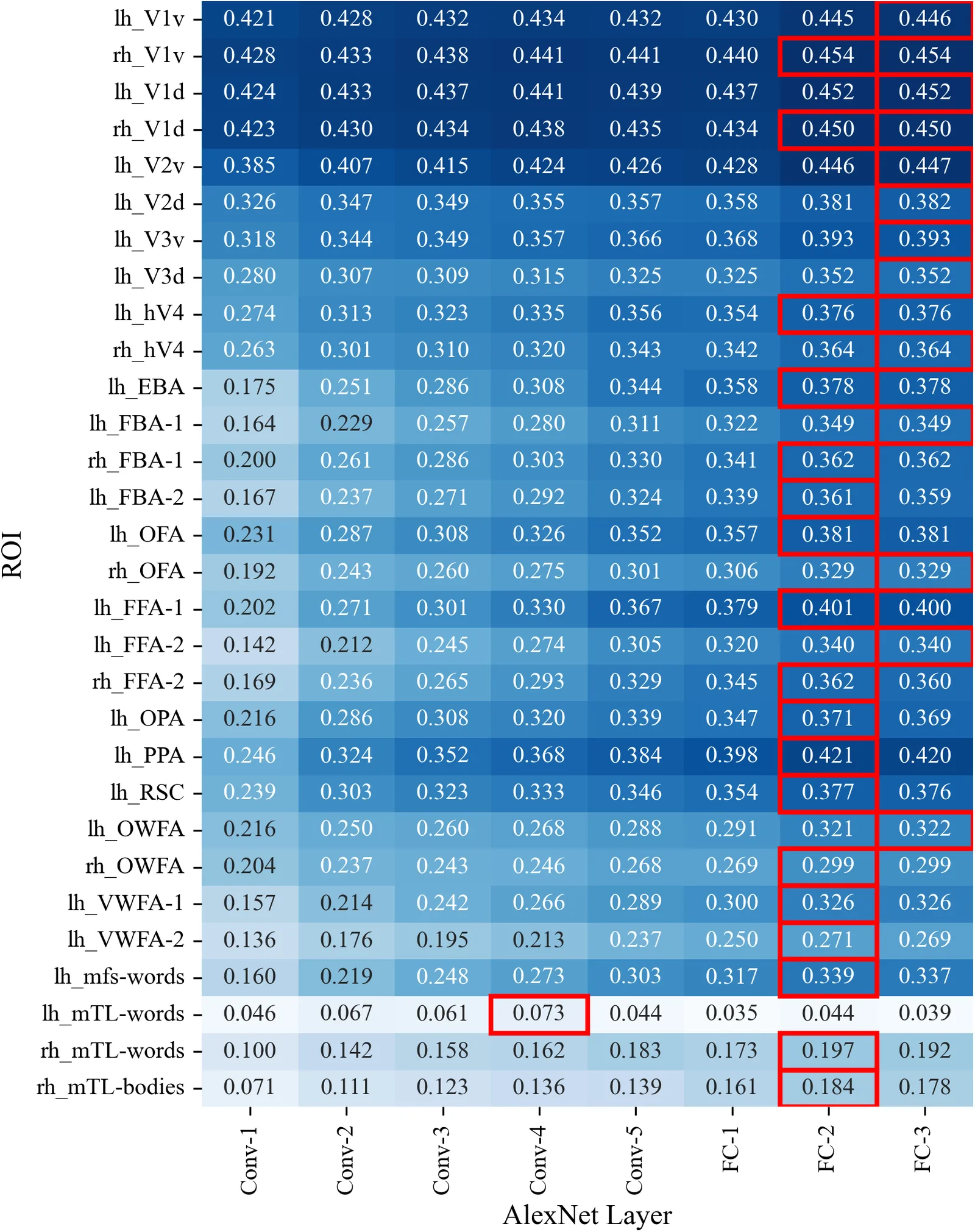
Regression-tuned AlexNet encoding scores per layer. Matrix of encoding scores averaged across subjects for each ROI and regression-tuned AlexNet layer, where the entries are the average test encoding accuracy over 5-fold cross validation when a particular AlexNet layer’s activations (after PCA for dimensionality reduction) are used to predict a particular ROI (using the best penalty term for each voxel and layer for ridge regression). Right hemisphere (rh) ROIs are omitted if they have the same best AlexNet layer as their corresponding left hemisphere (lh) ROI.

We also visualize the difference in encoding accuracy for each voxel across the cortex of each subject in Fig 3, where we use the FreeSurfer template [52]. Fig 4 shows which layers of the CL-tuned AlexNet models are most predictive of the voxels in each ROI, while Fig 5 shows which layers of the regression-tuned AlexNet models are most predictive for each ROI. In general, the CL fine-tuning causes the most predictive layer to be 1 or 2 layers later, while the regression-tuning makes the last or second-to-last layer the most predictive in almost all ROIs.

### Pooled subject fine-tuning

Pooling subjects while fine-tuning the AlexNet for a specific ROI creates a shared representation space that benefits from more data, and has the potential to improve the subsequent encoding for ROIs. The encoding performance results with pooled models (using an embedding dimension equal to the average number of voxels in the ROI across all subjects) versus subject-specific models, summarized in Table 3, suggest that there is higher similarity in visual representations across subjects in the early visual areas than in the higher visual areas. As compared to the subject-specific models, encoding performance is significantly improved when pooling in the early ROIs, but not significantly improved when pooling in the later ROIs. For the early ROIs, the corrected p-value is 0.004 and the effect size is 0.39 (Cohen’s d) for a paired, two-tailed t-test with Bonferroni correction for 2 tests and significance threshold of α=0.05; and for higher ROIs, the corrected p-value is 0.36 with an effect size of 0.06. As an ablation to test the effect of the size of the latent dimension, in S1 Table, we show the pooled versus subject-specific results when using an embedding dimension equal to the maximum number of voxels across all ROIs and subjects, which has minimal impact on the pooled results.

**Table 3:** Pooled versus subject-specific results for early (E) and higher (H) visual ROIs. For each subject, the average test set correlation *r* as described in Accuracy Metric across all early visual and higher visual ROIs is reported for CL-tuned AlexNet (Contrastive Learning for ROI-specific Feature Extraction) and pooled CL-tuned models (Pooled Models). The last two columns show the percentage of voxels, averaged across the early and higher visual ROIs, for which encoding with the pooled model was more predictive than the subject-specific model. Using a permutation test, lack of significance at a level of 0.01 is indicated by tilde above.

	Subj-Specific Avg. $\bar{r}$		Pooled Avg. $\bar{r}$		Pooled > Subj-Specific	
	E	H	E	H	E	H
1	0.477	0.403	0.501	0.399	61.9%	$\tilde{\text{44.2}}$%
2	0.461	0.436	0.483	0.426	64.7%	$\tilde{\text{32.7}}$%
3	0.385	0.350	0.415	0.357	83.7%	$\tilde{\text{51.0}}$%
4	0.391	0.342	0.405	0.354	80.8%	58.0%
5	0.467	0.474	0.458	0.463	$\tilde{\text{37.4}}$%	$\tilde{\text{27.5}}$%
6	0.385	0.314	0.402	0.329	79.1%	58.7%
7	0.379	0.331	0.408	0.342	85.3%	62.5%
8	0.327	0.262	0.351	0.271	86.3%	57.9%
	0.409	0.364	0.428	0.368	72.4%	49.1%

### External dataset validation

Table 4 contains the results of the external dataset validation experiment on the Natural Object Dataset [11], as described in External Dataset Validation. The ROI-specific feature extraction models fined tuned using CL on NSD’s Subject 1 improve the encoding prediction for voxels in the corresponding early ROIs in NOD. For all 9 subjects, more than half of the top 50 voxels per ROI with the highest signal to noise ratio in the early visual areas (V1, V2, V3, and V4) are better encoded (higher correlation) with the CL-tuned models. Overall, the average proportion of voxels improved is 70.6% and the average correlation increased from 0.142 to 0.153, an improvement of 8.1%. This supports the conclusion that the CL fine-tuning creates feature extraction models that process visual stimuli more similarly to the target ROI.

**Table 4:** Encoding performance for early visual ROIs (V1-V4) for subjects 1–9 in the Natural Object Dataset using feature extraction models tuned on NSD as described in External Dataset Validation. For each subject, the average test set correlation *r* as described in Accuracy Metric across all early visual ROIs is reported for untuned AlexNet and the AlexNet models tuned using CL pooled across subjects on NSD. The percentage of voxels for which the CL-tuned model was more predictive on average across the test set compared to the untuned AlexNet is also shown. Using a permutation test, lack of significance at a level of 0.01 is indicated by tilde above (threshold is 58.5% for all subjects).

	Avg. Ctrl. $\bar{r}$	Avg. CL $\bar{r}$	Avg. % of voxels improved vs. Ctrl.	CL vs. Ctrl. *r* Change
1	0.135	0.143	63.2	+5.4%
2	0.222	0.238	82.3	+7.0%
3	0.137	0.155	85.0	+13.3%
4	0.148	0.159	70.0	+7.5%
5	0.114	0.121	59.0	+5.7%
6	0.103	0.122	84.0	+18.2%
7	0.140	0.150	77.0	+6.6%
8	0.185	0.187	$\tilde{\text{48.0}}$	+1.1%
9	0.098	0.106	66.7	+7.9%
	0.142	0.153	70.6	+8.1%

The average encoding correlations for the NOD dataset are much lower than for the early visual ROIs in the NSD datasets (the best subject’s baseline is 0.222 in NOD versus 0.458 for the early visual ROIs in NSD). This is not unexpected due to three reasons: first, the scanner resolution for NOD is lower than NSD (3T instead of 7T); second, the responses to each image for NOD are only the result of a single trial rather than an average of 3 trials as in NSD; and third, NOD has fewer responses used for fitting the linear models than NSD (3200 instead of approximately 9000 per subject). However, the relative encoding performance for different methods still provides a meaningful metric for how well each feature extraction model is tuned for that ROI. Because the CL–tuned models were fine-tuned on a completely different dataset, the improved encoding accuracy for the brain-tuned models over the untuned models indicates that fine-tuning on NSD generalizes to other datasets.

### Downstream classification tasks

**Fig 6:**
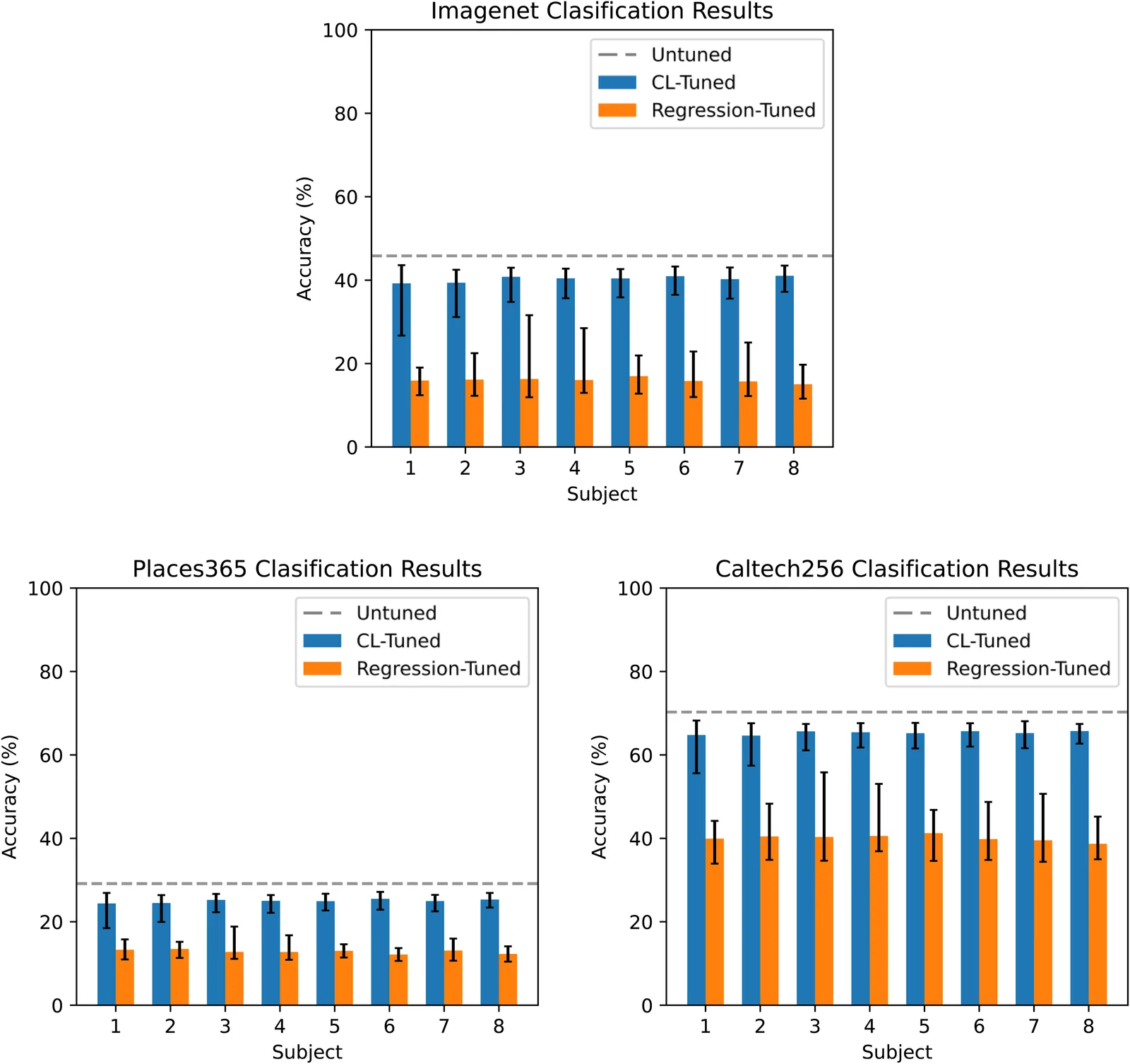
Downstream classification task results. Results for baseline and brain-tuned AlexNet on the validation portion of ImageNet ILSVRC2012 (the training portion was used for the pretraining the AlexNet baseline), Caltech256, and Places365. The bar height is the average accuracy across all ROIs tuned using that method for that subject. Error bars represent the full range of results across all ROIs (minimum to maximum).

Using the methodology from Downstream Classification Tasks we evaluate the test set classification accuracy for image classifiers built on top of the untuned, subject-specific CL-tuned and regression-tuned models for the ImageNet ILSVRC2012, Caltech256, and Places365 datasets. Fig 6 summarizes the performance of the subject specific tuned models versus the untrained baseline. See S5-S7 Figs for the classification accuracy per ROI for both the subject-specific and pooled models for each ROI.

For both the ImageNet classification task and the non-training classification tasks (Caltech256 and Places365), we see a drop in classification accuracy when using the brain-tuned models, but the CL-tuned models suffer much less of a decrease than the regression-tuned models. For ImageNet, the baseline pretrained on ImageNet achieves 45.8% accuracy. Across all possible subject-ROI pairs, the best CL model achieves a test accuracy of 43.6% (LH EBA in subject 1), see S5 Fig. This means there is only a 1.6% drop in performance after tuning to brain-activity (while starting from pretrained). For Places365, the most challenging task, the baseline pretrained model achieves 29.1% accuracy. Across all possible subject-ROI pairs, the best CL model achieves a test accuracy of 27.2% (LH OPA and LH PPA in subject 6), see S6 Fig. For Caltech256, the baseline pretrained model achieves 70.3% accuracy. Across all possible subject-ROI pairs, the best CL model achieves a test accuracy of 68.2% (LH EBA in subject 1), see S7 Fig. These results show that on average, the CL-tuning preserves most of the information content necessary for using the image features of the CNN for image classification tasks, especially in task-relevant ROIs. Quantitatively, the values are consistent across subjects.

### Representational similarity analysis

**Fig 7:**
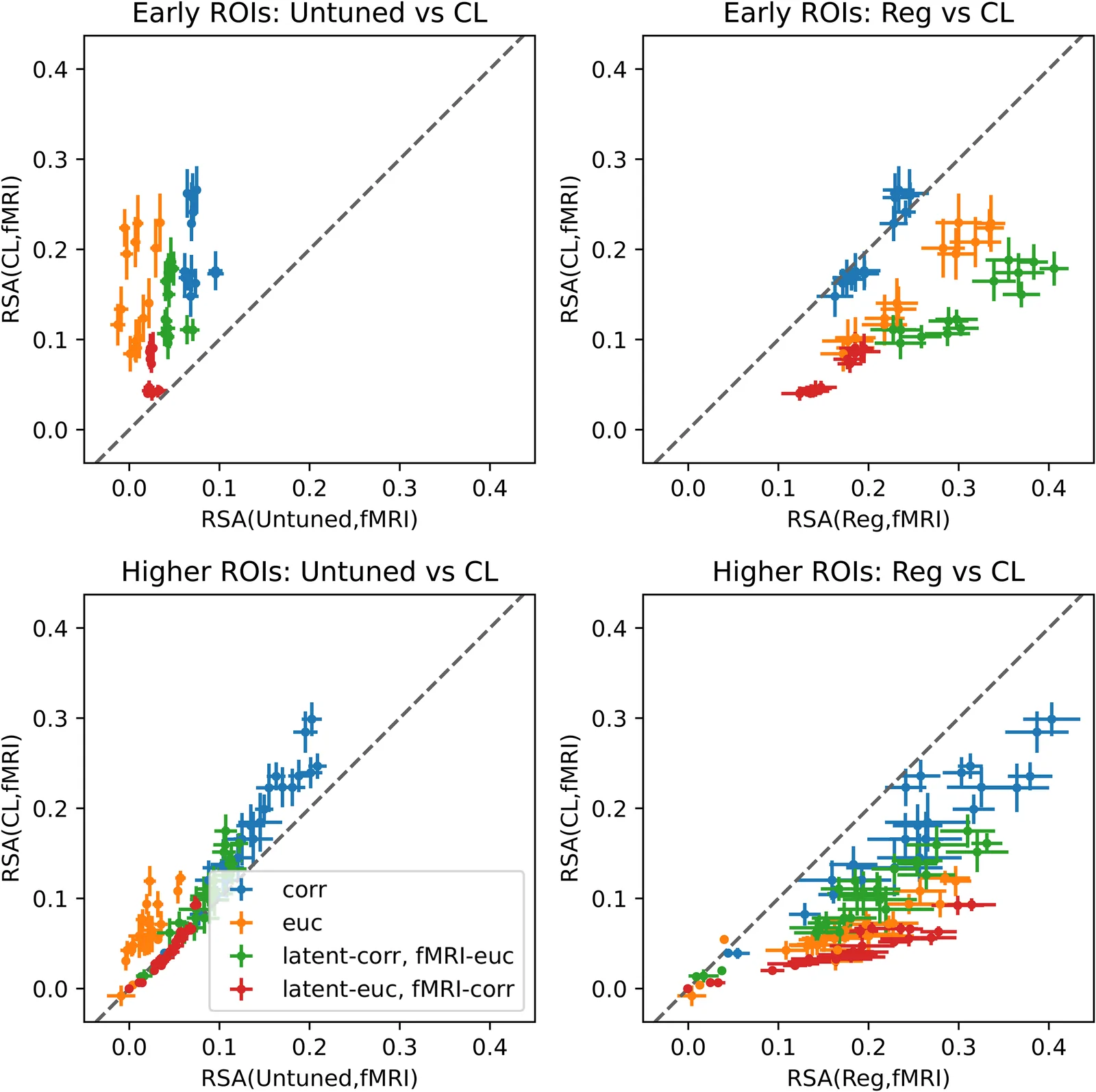
RSA between fMRI to model. Scatter plot comparing the representational similarity analysis (Spearman’s ρ using either correlation distance (corr) or Euclidean distance (euc) as the dissimilarity measures) between each ROI’s fMRI response and the untuned AlexNet model’s representation, CL fine-tuned model, or regression-tuned (Reg) model. Error bars indicate standard errors across the subjects.

While the Bhattacharya-based dissimilarity metric allows for direct comparisons of the classification models, RSA allows for direct comparisons of representations (brain or network based). At the individual subject level we use the held out test set (15% of the dataset with sample size ranging from 1317–1477) to compare each ROI’s CL-tuned model’s representation (both the full network *f* and the truncated $f_{l}$, where *l* is the best layer selected for the encoding model) directly to the fMRI BOLD response (beta coefficients). We do the same for the untuned AlexNet representations and the regression-tuned representations (both full network and truncated). The results for the full networks are shown in Fig 7. Additionally, we compare pretrained AlexNet to CLIP vision transformer, ViT-L-14 in S4 Fig, finding that there is generally lower RSA compared to untuned AlexNet when using correlation on the representation, but slightly higher RSA when using Euclidean dissimilarity.

The results show that the CL-tuned models have higher RSA with the fMRI responses than the untuned AlexNet model, especially as measured by the correlation distance. For early ROIs, the RSA are the same or slightly higher for the CL-tuned compared to regression-tuned using the correlation distance; however, using the Euclidean distance for dissimilarity yields higher RSA for the regression tuned. This is logical since the CL training ignores the norm of the embeddings, while for regression it is important. For the higher (later) ROIs, there are still gains for CL-tuned as compared to untuned, but regression-tuned representations have higher RSA for both correlation and Euclidean distance.

**Fig 8:**
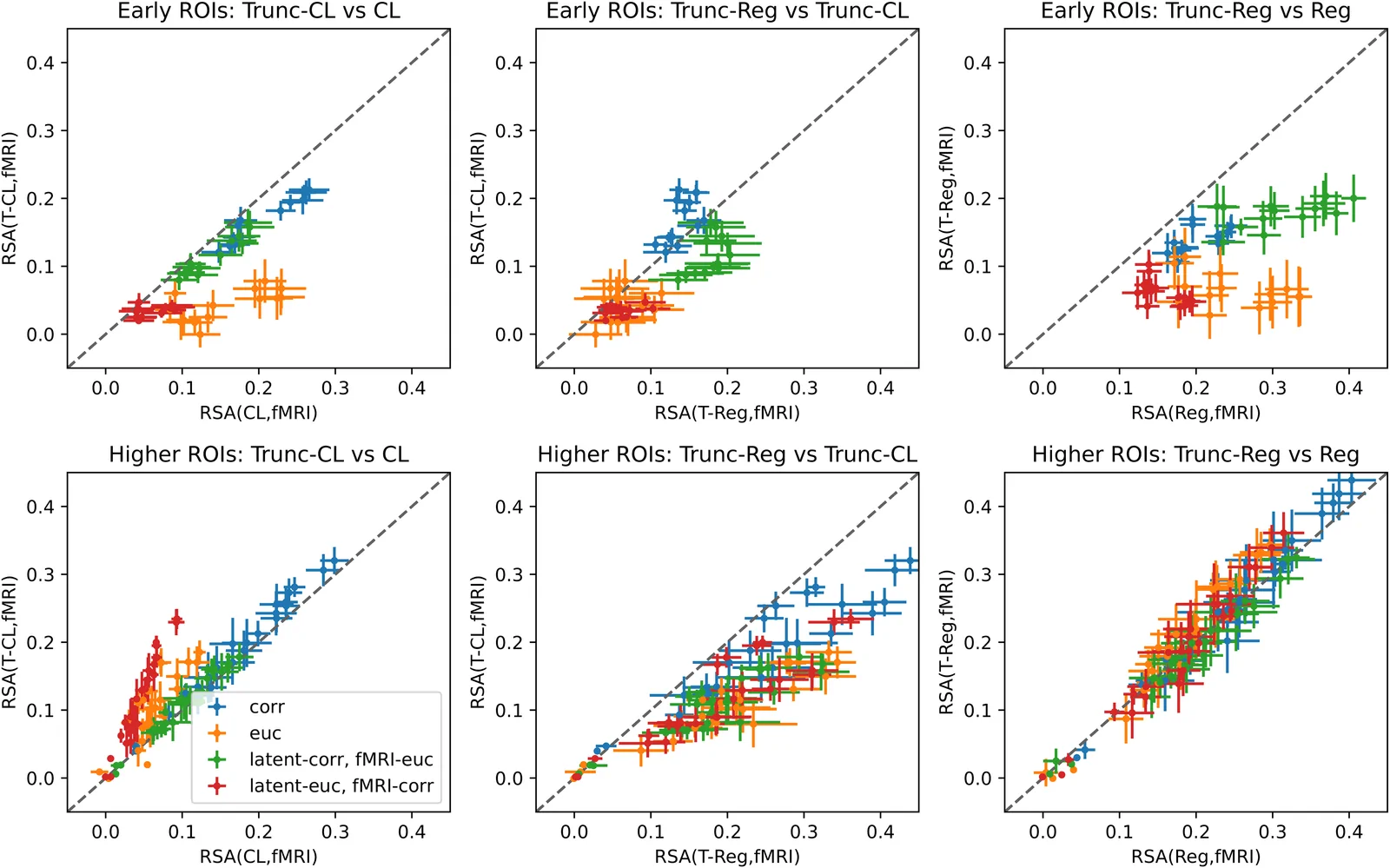
RSA between fMRI to truncated models. Scatter plot comparing the representational similarity analysis (Spearman’s ρ using either correlation distance (corr) or Euclidean distance (euc) as the dissimilarity measures) between each ROI’s fMRI response and the CL fine-tuned model, regression-tuned (Reg) model, or the corresponding network truncated at the layer used for the encoding model (Trunc-CL/T-CL and Trunc-Reg/T-Reg). Error bars indicate standard errors across the subjects.

Fig 8 shows that the truncated representations, which use the best output layer for the encoding model, generally have lower RSA than the full model. This makes sense because there may be additional variation in the earlier layers that is then filtered out by the encoding weights. One exception is the truncated CL-tuned and regression-tuned models in higher (later) ROIs. In early ROIs, the RSA values with the fMRI are higher for the truncated CL-tuned compared to the truncated regression-tuned. This matches the encoding performance, for which CL outperformed regression in early ROIs.

**Fig 9:**
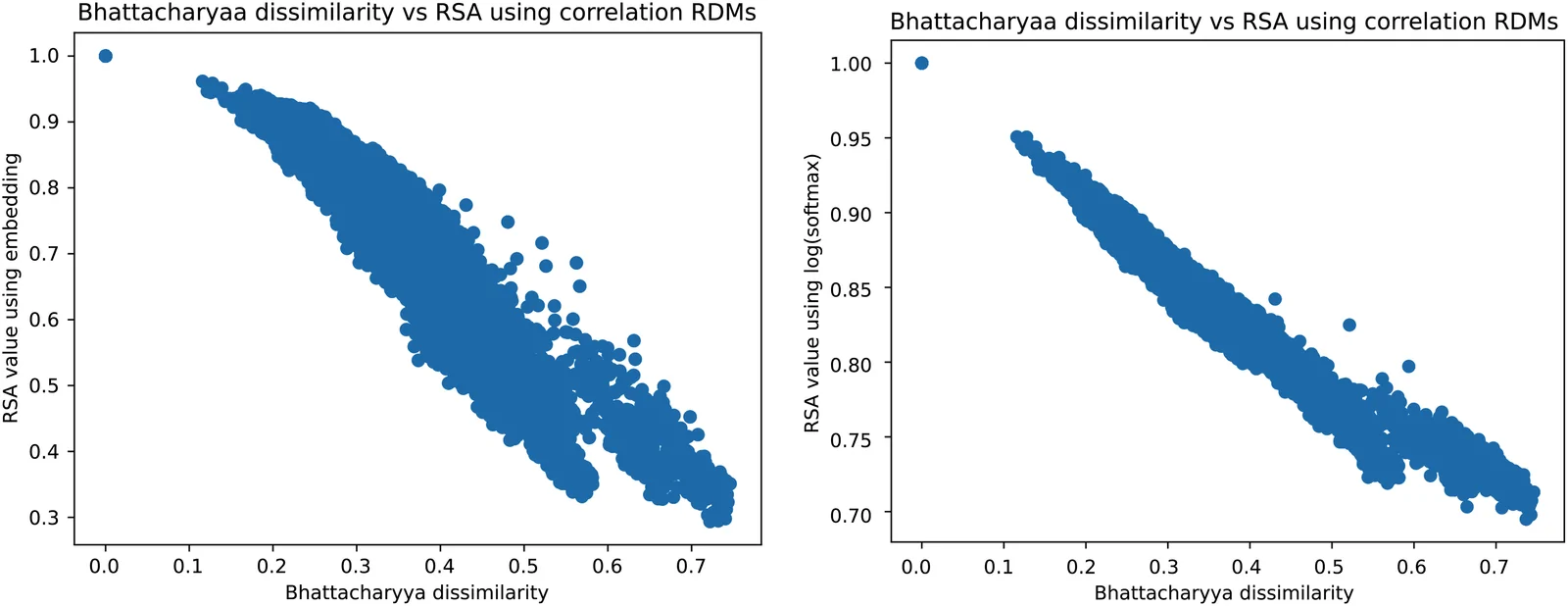
Bhattacharyya-based dissimilarity compared to RSA. Scatter plots comparing the Bhattacharyya-based dissimilarity and RSA between all pairs of CL-tuned model across all subjects and ROIs.

We also compare the representations among subjects on a subset of 1000 of the images from ImageNet that were used to test the downstream classifiers. We directly compare the Bhattacharyya-based dissimilarity to RSA of the CL-tuned network’s representation created by *f* in Fig 9. The results show that dissimilarity based on the classification head trained for ImageNet classes correlates with RSA for the correlation distance.

Table 5 details the statistics of the RSA values between ROI-specific CL-tuned (and regression-tuned) representations for different subjects. The models for early ROIs have lower intersubject RSA compared to the models for higher (later) ROIs. While the regression-tuned representations appear more subject specific, the RSA values must be contextualized by the inter-ROI RSA values. To quantify this we calculate a test statistic (after first performing the Fisher transformation on the similarity values) that is the difference between average intersubject RSA within-ROI and the average inter-ROI RSA, using a permutation test of the ROI labels per subject to generate a surrogate distribution using 2000 permutations, and report the p-value in the table. The results for the two tuning methods match almost exactly, with the CL-tuning also indicating that V2d has more similarity between subjects than between ROIs. The ROIs with the lowest p-values are all higher (later) ROIs.

**Table 5:** Intersubject RSA values. Statistics of intersubject RSA values among N≤8 subjects for the CL-tuned models (top) and Reg.-tuned models (bottom) across ROIs using correlation distance on the log-softmax of 1000 ImageNet images. Mean, standard deviation, and min-max are across the *N* choose 2 unique pairs. The p-value is for the difference between average within-ROI, intersubject RSA and the average inter-ROI RSA, and is calculated by a permutation the ROI labels per subject across 2000 runs, with bold indicating significance at a level of 0.05.

ROI	Left Hemisphere				Right Hemisphere			
	N	Mean ± SD	(Min–Max)	p-value	N	Mean ± SD	(Min–Max)	p-value
	CL-tuned							
V1v	8	0.83±0.03	(0.78–0.87)	0.0770	8	0.84±0.02	(0.81–0.88)	**0.0095**
V1d	8	0.82±0.04	(0.76–0.86)	0.1450	8	0.83±0.03	(0.78–0.87)	0.1910
V2v	8	0.85±0.01	(0.83–0.87)	**0.0025**	8	0.85±0.02	(0.81–0.88)	**0.0120**
V2d	8	0.86±0.01	(0.84–0.88)	**0.0480**	8	0.86±0.01	(0.84–0.88)	**0.0380**
V3v	8	0.87±0.01	(0.86–0.89)	**0.0050**	8	0.87±0.01	(0.84–0.89)	**0.0230**
V3d	8	0.88±0.01	(0.86–0.89)	**0.0460**	8	0.88±0.01	(0.85–0.90)	**0.0170**
hV4	8	0.88±0.01	(0.87–0.89)	**0.0170**	8	0.87±0.01	(0.85–0.89)	0.0535
EBA	8	0.93±0.00	(0.92–0.94)	**0.0000**	8	0.93±0.00	(0.92–0.94)	**0.0000**
FBA-1	5	0.90±0.00	(0.89–0.90)	**0.0210**	6	0.90±0.01	(0.88–0.91)	**0.0100**
FBA-2	7	0.89±0.01	(0.88–0.90)	**0.0020**	8	0.89±0.01	(0.88–0.90)	**0.0000**
mTL-bodies	–	–	–	–	2	0.90	–	**0.0210**
OFA	8	0.89±0.01	(0.88–0.90)	**0.0230**	8	0.89±0.01	(0.88–0.90)	**0.0065**
FFA-1	8	0.89±0.00	(0.89–0.90)	**0.0025**	8	0.90±0.01	(0.88–0.91)	**0.0005**
FFA-2	7	0.89±0.01	(0.87–0.90)	**0.0055**	6	0.89±0.00	(0.89–0.90)	**0.0050**
OPA	8	0.91±0.00	(0.91–0.92)	**0.0000**	8	0.91±0.00	(0.90–0.91)	**0.0000**
PPA	8	0.91±0.00	(0.90–0.91)	**0.0000**	8	0.90±0.01	(0.89–0.92)	**0.0000**
RSC	8	0.89±0.01	(0.88–0.90)	**0.0005**	8	0.89±0.01	(0.88–0.90)	**0.0000**
OWFA	8	0.89±0.01	(0.87–0.90)	**0.0230**	8	0.89±0.01	(0.87–0.90)	**0.0250**
mfs-words	8	0.89±0.00	(0.88–0.91)	**0.0010**	6	0.89±0.01	(0.88–0.90)	**0.0180**
	Regression-tuned							
V1v	8	0.58±0.03	(0.54–0.68)	0.0780	8	0.56±0.03	(0.52–0.66)	**0.0060**
V1d	8	0.58±0.04	(0.52–0.68)	0.1560	8	0.56±0.04	(0.50–0.68)	0.1690
V2v	8	0.59±0.03	(0.55–0.67)	**0.0045**	8	0.58±0.03	(0.54–0.67)	**0.0090**
V2d	8	0.57±0.03	(0.52–0.66)	0.0540	8	0.59±0.02	(0.54–0.62)	**0.0435**
V3v	8	0.57±0.02	(0.53–0.64)	**0.0075**	8	0.58±0.03	(0.54–0.68)	**0.0195**
V3d	8	0.57±0.02	(0.55–0.64)	**0.0350**	8	0.59±0.02	(0.57–0.66)	**0.0135**
hV4	8	0.58±0.02	(0.55–0.64)	**0.0135**	8	0.58±0.02	(0.55–0.65)	0.0575
EBA	8	0.63±0.02	(0.59–0.68)	**0.0000**	8	0.60±0.02	(0.56–0.64)	**0.0000**
FBA-1	5	0.61±0.02	(0.58–0.66)	**0.0220**	6	0.59±0.02	(0.55–0.62)	**0.0065**
FBA-2	7	0.59±0.02	(0.54–0.64)	**0.0010**	8	0.63±0.03	(0.58–0.68)	**0.0010**
mTL-bodies	–	–	–	–	2	0.68	–	**0.0200**
OFA	8	0.58±0.03	(0.52–0.64)	**0.0180**	8	0.58±0.03	(0.49–0.64)	**0.0085**
FFA-1	8	0.61±0.02	(0.56–0.63)	**0.0025**	8	0.62±0.02	(0.59–0.68)	**0.0010**
FFA-2	7	0.60±0.04	(0.53–0.68)	**0.0035**	6	0.62±0.02	(0.59–0.65)	**0.0085**
OPA	8	0.57±0.03	(0.50–0.62)	**0.0000**	8	0.57±0.02	(0.52–0.61)	**0.0000**
PPA	8	0.58±0.01	(0.55–0.60)	**0.0000**	8	0.58±0.02	(0.55–0.62)	**0.0000**
RSC	8	0.60±0.03	(0.55–0.65)	**0.0000**	8	0.57±0.02	(0.54–0.61)	**0.0005**
OWFA	8	0.56±0.03	(0.49–0.60)	**0.0290**	8	0.55±0.03	(0.48–0.62)	**0.0250**
mfs-words	8	0.64±0.04	(0.59–0.74)	**0.0000**	6	0.63±0.02	(0.58–0.66)	**0.0165**

### Model landscapes

To understand the similarity of the classification models trained for the ImageNet dataset on top of the feature extraction models we apply multidimensional scaling (MDS) of the Bhattacharyya-based dissimilarity metric [8] between the prediction vectors from the downstream classifier for ImageNet for each model. When we apply this to the full set of models (untuned AlexNet, CL-tuned for each subject and ROI, CL-tuned pooled subject for each ROI, and regression-tuned for each subject and ROI), we find that each group of models is distinct, as shown in S8 Fig. In comparison, models landscapes with different subsets of the models will use different components of the dissimilarity space as the axes of model embedding coordinates.

**Fig 10:**
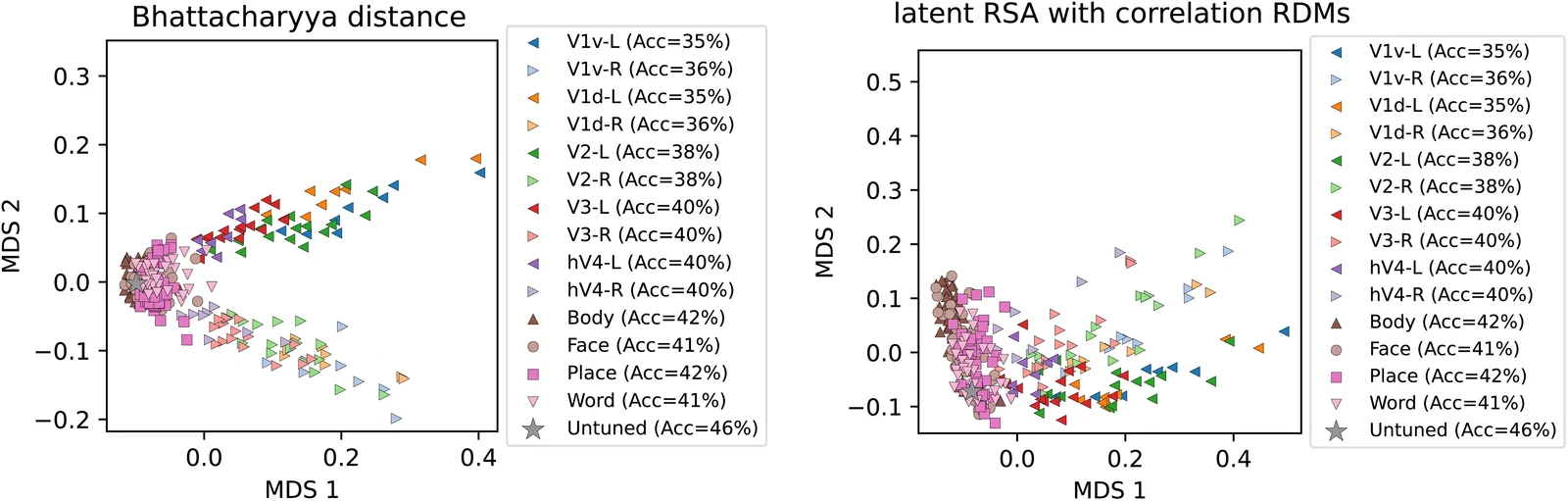
Overall ROI model landscapes. Model landscapes for subject-specific ROI-specific CL fine-tuned models and untuned AlexNet using Bhattacharyya dissimilarity based on classification head (Left) or RSA with correlation distance (Right). The early visual ROIs are separated from from higher ROIs along the first dimension (MDS 1) and early ROIs separate into left and right along the second dimension (MDS 2).

Fig 10 shows the landscape when taking only the untuned AlexNet and subject- and ROI-specific CL fine-tuned models. Models tuned with CL for different subjects, but the same ROI, appear in the same arrangement and are relatively similar based on the their embedding coordinates. The landscape is arranged such that the first axis separates early versus late ROIs, but the second axis separates the left and right hemispheres in the early ROIs (V1–4). This indicates that predictions for left and right ROI models are systematically different across the images—due to the lateralized presence of objects related to the classification task and the retinotopic tuning in V1–4. Higher level (late) ROIs have more similar predictions to untuned AlexNet predictions, and are more accurate.

**Fig 11:**
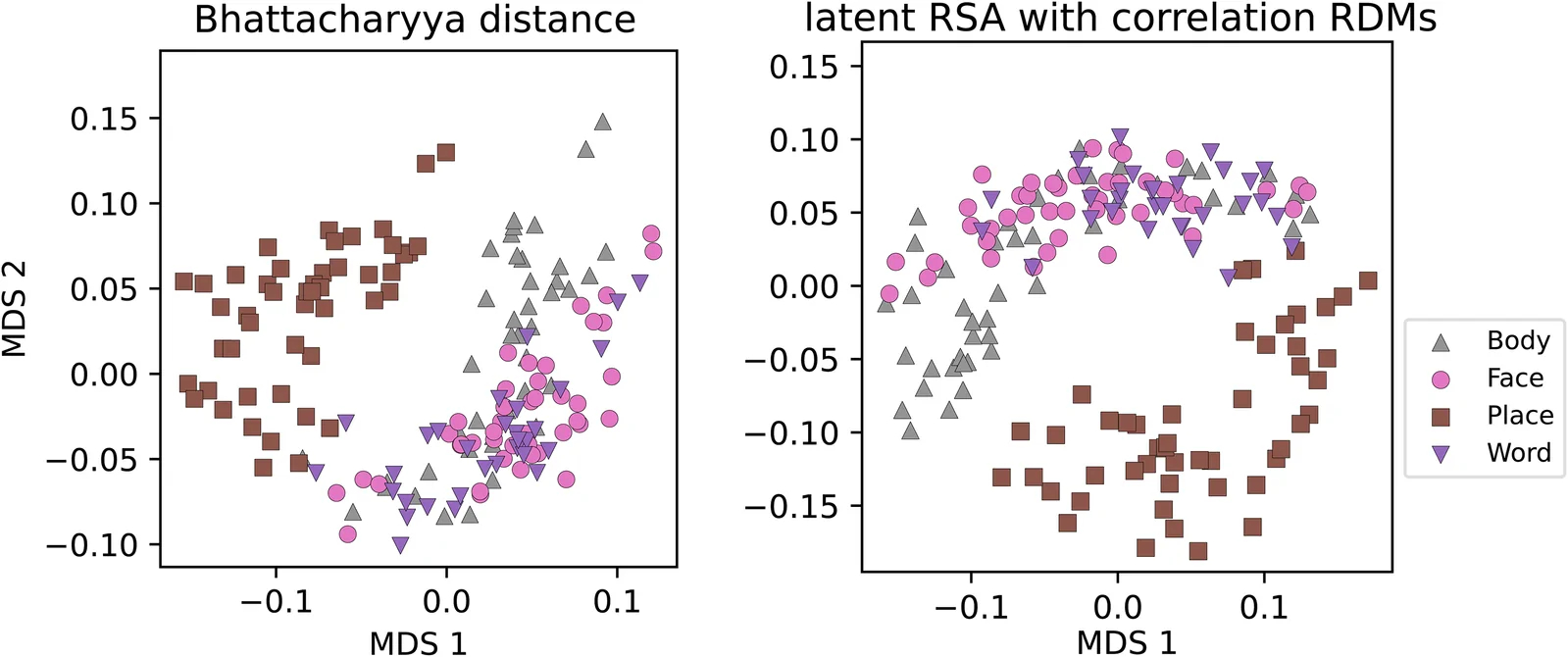
Higher visual ROI model landscapes. Model landscape for higher level (late) subject-specific ROI-specific fine-tuned models.

Fig 11 shows the landscape when taking only the untuned AlexNet and subject- and ROI-specific CL fine-tuned models for higher level ROIs. Here we see the two axes separate the models by ROI group related to representation of body, face, place, and words. The ROIs associated to face and place are generally worse for classification, but clearly distinct in this embedding. Again, models tuned for different subjects but the same ROI appear in the same arrangement and are relatively similar based on the their embedding coordinates. Notably, the untuned AlexNet is not most similar to the highest performing fine-tuned model: subject 1’s EBA (left hemisphere), as detailed in S5 Fig. This reveals that fine-tuned models may have deviations in the predictions from the untuned AlexNet while still retaining features useful for downstream classification tasks.

**Fig 12:**
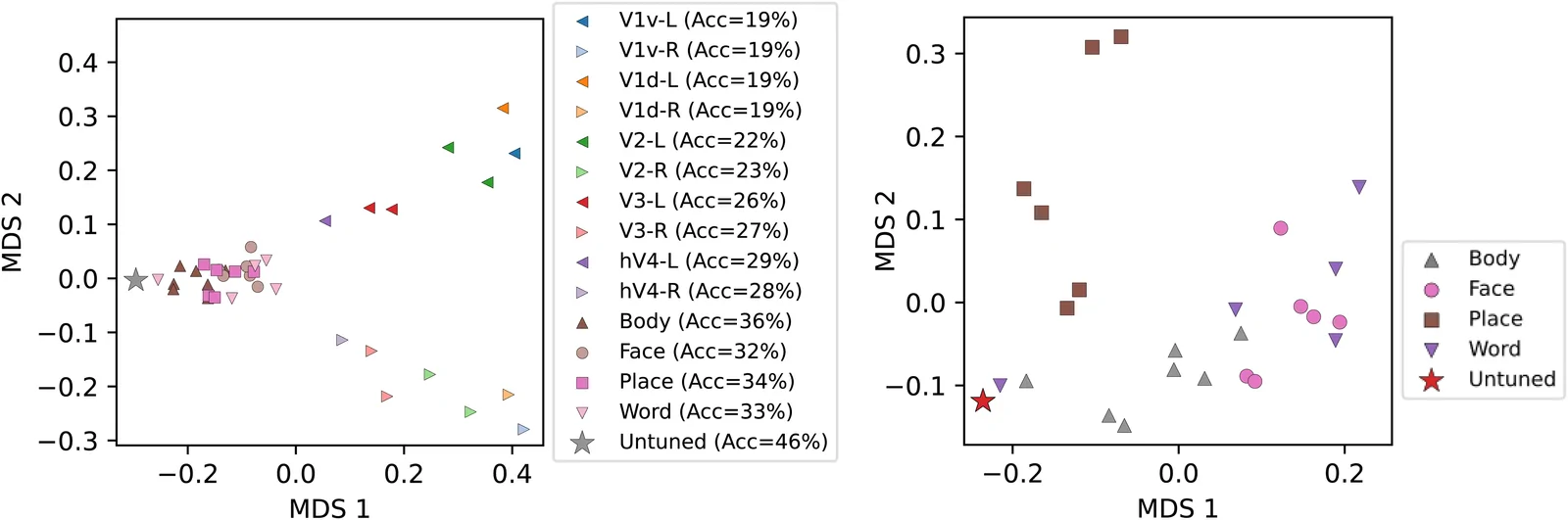
Model landscapes with pooled-subject CL-tuning. Model landscapes for untuned AlexNet (star marker) and pooled-subject ROI-specific ROIs. Left plot is all ROIs and right plot is higher level (late).

Fig 12 shows that the landscape which only embeds the untuned AlexNet and pooled-subject CL-tuned models matches the landscape for the subject-specific CL fine-tuning.

**Fig 13:**
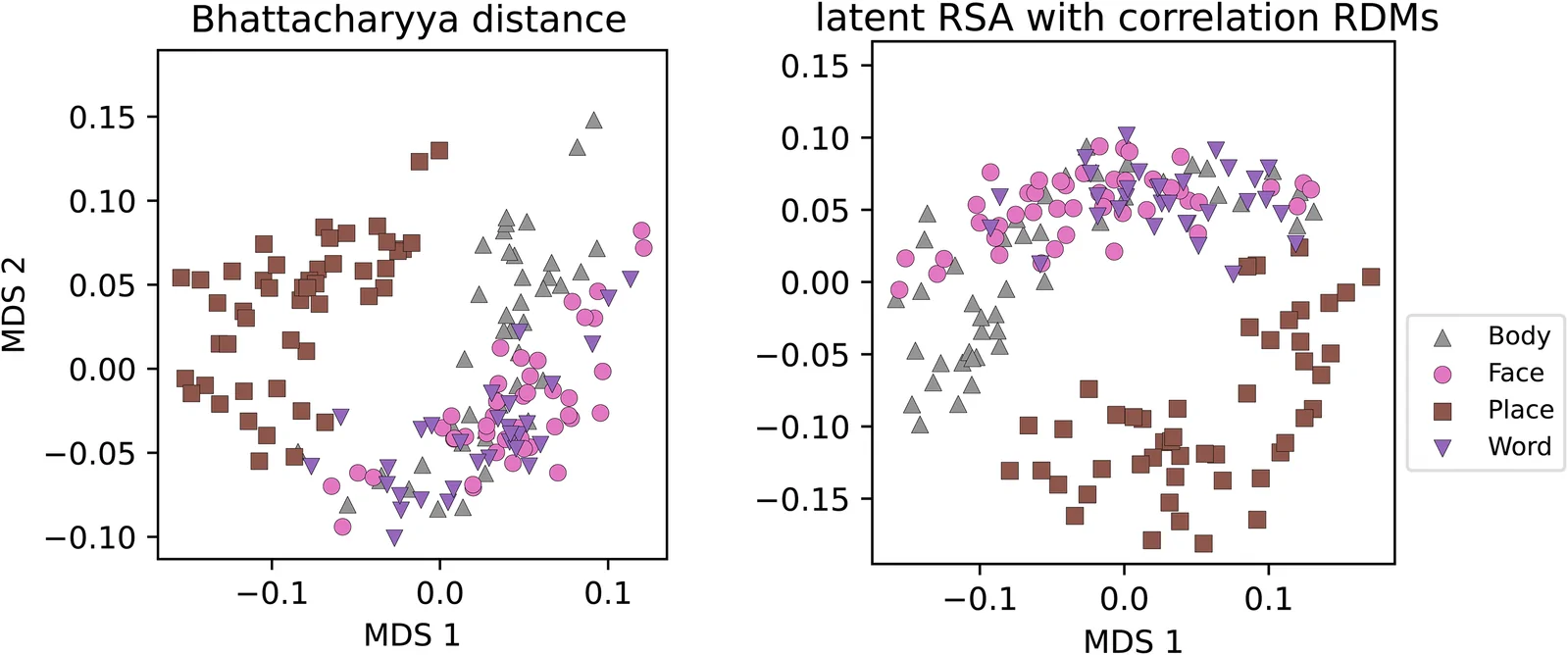
Regression-tuned model landscapes. Model landscape for subject-specific and ROI-specific regression fine-tuned models and AlexNet (star marker). Left plot is all ROIs and right plot is higher level (late).

Finally, we note that the landscape for the regression-tuned, subject-specific and ROI-specific models differs from the CL-tuned model landscapes, as shown in Fig 13. Notably, while the ROIs of different subjects are similar and the primary axis of dissimilarity is associated with early versus later ROIs, the second axis separates place and face ROIs in the later areas. This indicates that the regression tuning yields a different landscape. Looking at only the later ROIs, there is less clustering than in the CL tuning. To quantify this, we compute the average silhouette index for the distances in the 2D MDS landscapes for different ROI groupings and a jackknife confidence interval for each. The results in Table 6 show that the regression-tuned Silhouette score is lower but still inside the 95% CI of the CL-tuned for higher-only. However, using fine-grained ROI labels and grouping higher ROIS the CL-tuning is better and the regression-tuned is outside the CI. Using a coarse grouping of early layers, regression-tuned has higher score and CL is outside the CI. Finally, there is not much difference for early versus late (which has the highest Silhouette scores for both).

**Table 6:** Silhouette scores for ROI groups in landscapes. Four different ROI groupings are explored. Jackknife computed by leaving out one subject.

ROI Grouping	Method	Mean (Jackknife 95% CI)
Early vs. higher (2) {Early,Higher}	CL Reg.	0.542 (0.512–0.572) 0.529 (0.486–0.572)
Fine-grained early and late grouped (11): {V1v-L,V1v-R,V1d-L,..., hV4-R, Higher}	CL Reg.	0.308 (0.261–0.355) 0.055 (-0.009–0.119)
Higher only (4) {Body, Face, Place, Word}	CL Reg.	0.110 (0.007–0.213) 0.051 (-0.069–0.171)
All coarse (5) {Early, Body, Face, Place, Word}	CL Reg.	0.018 (-0.014–0.050) 0.229 (0.148–0.310)

### Generating stable diffusion images from CL-tuned models

**Fig 14:**
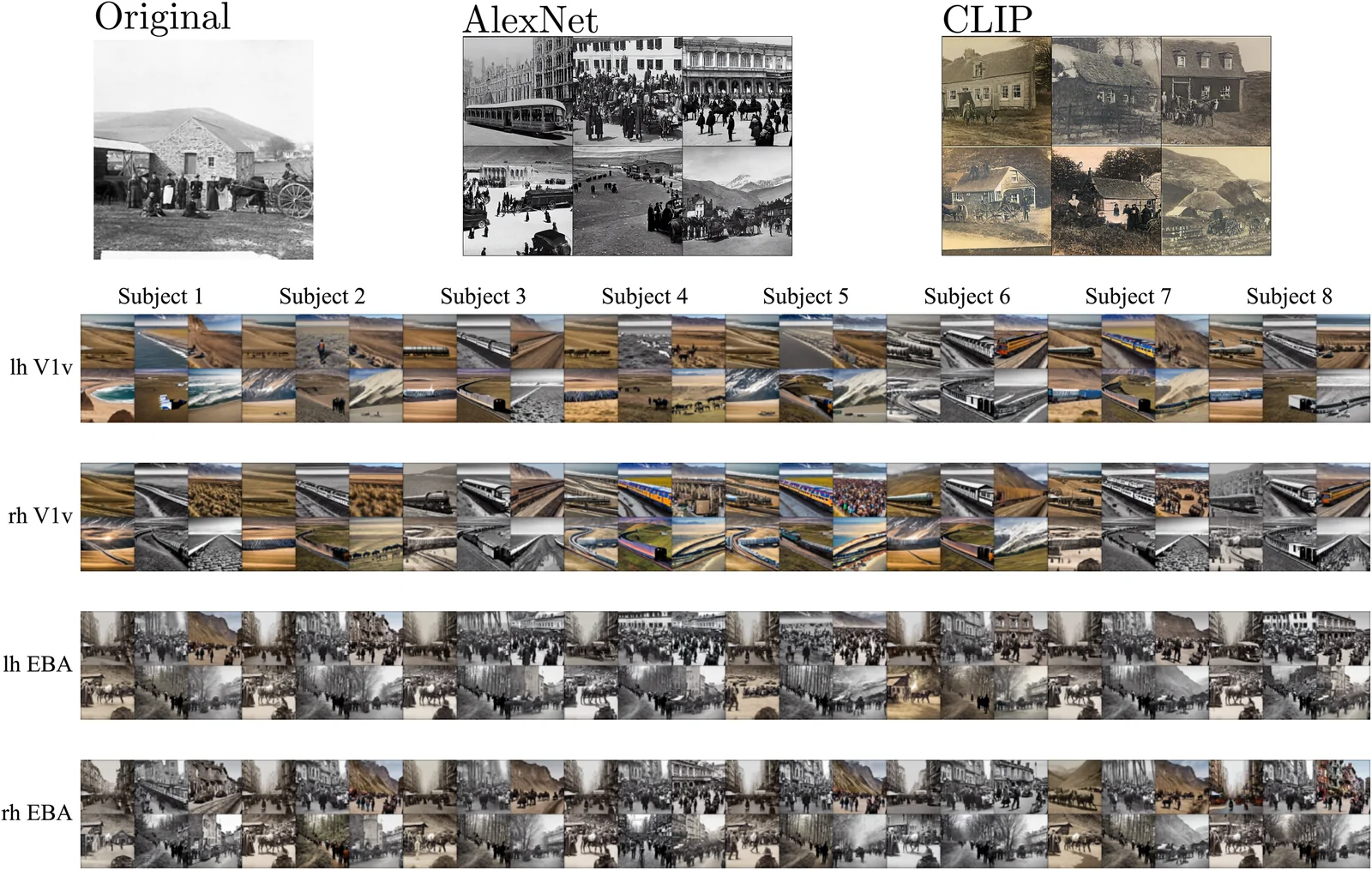
Image-to-Prompt Generative Examples. The shared prompt image (a center cropped image from John Thomas, The National Library of Wales, Creative Archive Licence) are shown with 6 generations of Stable Diffusion for untuned AlexNet, CLIP, and 32 subject-ROI specific CL-tuned models (8 subjects and left/right hemisphere V1v and EBA).

To illustrate how the CL-tuned models can reveal visual processing of different visual ROIs, we visualize the Stable Diffusion output for the CL-tuned models for left and right hemisphere V1v and EBA for all 8 subjects for a single prompt image in Fig 14. We find that there are distinct differences in the output for V1v and EBA which are consistent across subjects. Whereas the images generated from the EBA-tuned models feature close-ups of people or groups of people (where their faces are visible), the images generated from the V1v-tuned models mostly contain people riding motorcycles, bicycles, or doing other sports, without clearly visible faces. The similarity across subjects and distinctiveness between the outputs from V1v and EBA indicate the the CL fine-tuning induces changes in processing that can be used for “in-silico” experiments with the ROI-tuned models.

**Fig 15:**
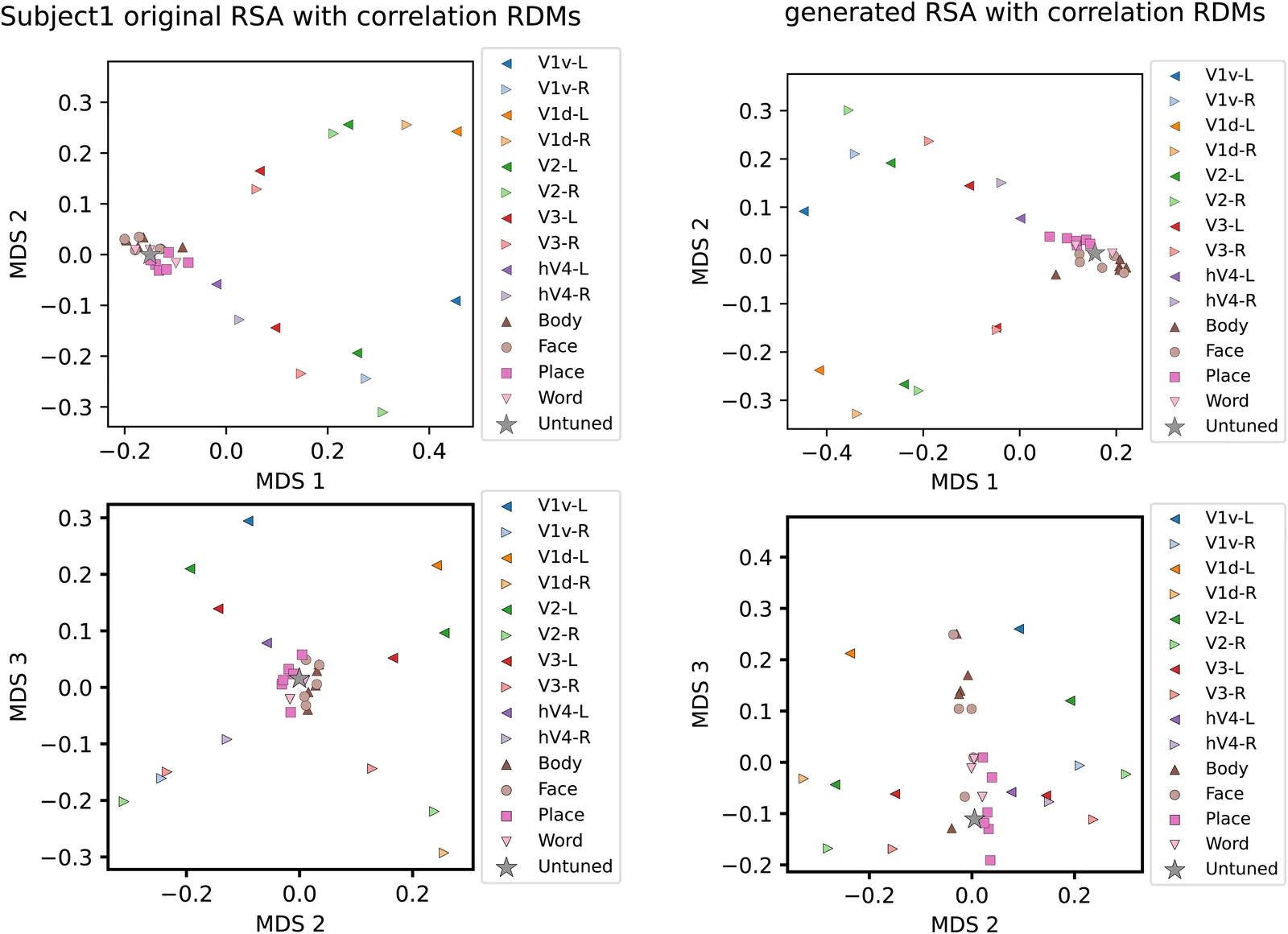
Subject 1 ROI model landscapes with Generative AI. Model landscapes for the Subject 1’s ROI-specific CL fine-tuned models using RSA with correlation distance using the original embeddings (Left) compared to embeddings of images generated with StableDiffusion (Right). The early visual ROIs are separated from from higher ROIs along the first dimension (MDS 1) and early ROIs separate into ventral and dorsal along the second dimension (MDS 2), and left and right along the third dimension (MDS 3).

To validate the cyclic consistency of the in-silico framework, for each of Subject 1’s CL-tuned ROI models (and the untuned AlexNet) we generate 1000 images using the CLIP-aligned embeddings of the 1000 images from ImageNet as prompts. We then embed them using the corresponding model. Ideally, for each prompt image embedding, the corresponding generated image embedding would be the closest, whereas a random permutation would have the generated image as the 500th nearest image. We use rank of the distance to the nearest neighbor (using correlation distance) to quantify cyclic consistency for each ROI. We find that the average ranks (denoted $\bar{k}$) are generally low (31.5–123) across the ROIs, compared to 30.6 for AlexNet and 11.7 for CLIP (ViT-L-14) (CLIP provides a performance bound). We also apply RSA to compare how similar the dissimilarity is between the original images and the generated images, with values ranging from 0.44–0.74 compared to 0.76 for AlexNet and 0.45 for CLIP. Both quantitative measures are in Table 7. The lower RSA for CLIP is unexpected, as generally the two measures agree across subject 1’s 33 ROIs (Spearman rho of -0.841). To understand the topology of the embeddings of the original versus generated images, we also estimate the intrinsic dimensionality (ID) using the TwoNN [18] method implemented in scikit-dimension [53] using the original images and the generated ones. The values, shown in Table 7, indicate that the generated image representations have a lower ID estimate, indicating that using the embeddings for conditioning and the generating process produces images that could be described as lying on a lower dimensional manifold. Nonetheless, across the ROIs, ID estimates of the original and generated latent representations correlate (Spearman rho of 0.718). AlexNet’s ID estimates are 17.8 for the original and 11.3 for the generated. CLIP’s ID estimates are 18.3 and 16.4, respectively. This is the highest ID for the generated images and the lowest gap of 1.9, confirming that CLIP preserves the most information through the generative process, but that the generated images exist on a lower-dimensional manifold than the original images. The higher ID for CLIP could explain why its RSA is lower, since there is more variety in the generated images, but Stable Diffusion was originally trained with text embedding conditioning. Thus, while the relative topological arrangement is preserved for CLIP (small $\bar{k}$) there is a distributional shift after generation, which the CLIP embedding is sensitive to. In contrast the ROI-tuned embeddings are tuned more “narrowly”, filtering out variation created by Stable Diffusion. Furthermore, it is notable that the ID for many ROI-tuned models is higher than CLIP or AlexNet, indicating that they preserve more information about the original image, which is subsequently lost by the Stable Diffusion generation. In Fig 15, we also show that the model landscape derived from the RSA representations of the generated images recapitulates aspects of the subject-specific landscape. We note that for this subject, the second dimension of the landscape captures dorsal-ventral differences and the third dimension corresponds to left-right lateralization.

**Table 7:** Cyclic Consistency of Image-to-Prompt Generative Images. The average rank ($\bar{k}$) of corresponding generated image (lower is better: 1 is ideal and 500 is chance), RSA values (higher is better: 1 is ideal) using original versus generated images, and the intrinsic dimensionality (ID) estimates using TwoNN [18] of original and generated representations across ROIs.

ROI/Model	Left Hemisphere				Right Hemisphere			
	$\bar{k}$	RSA	ID Orig.	ID Gen.	$\bar{k}$	RSA	ID Orig.	ID Gen.
V1v	96.9	0.57	16.1	10.2	69.8	0.54	21.6	14.7
V1d	99.3	0.52	14.4	9.8	122.9	0.44	19.9	14.0
V2v	68.9	0.59	18.8	11.8	107.3	0.51	21.5	13.3
V2d	68.5	0.55	22.0	14.8	74.5	0.55	21.4	14.6
V3v	55.4	0.63	22.4	14.5	63.1	0.57	20.7	15.2
V3d	44.0	0.64	21.6	15.2	42.9	0.66	22.2	14.7
hV4	37.7	0.66	22.8	14.7	41.7	0.65	20.6	14.5
EBA	33.2	0.76	16.8	11.0	30.7	0.74	18.7	11.4
FBA-1	32.0	0.72	20.7	14.4	36.9	0.68	22.3	15.7
FBA-2	–	–	–	–	38.1	0.72	20.0	13.6
OFA	36.5	0.67	21.5	14.7	33.9	0.66	21.0	15.1
FFA-1	31.5	0.70	21.2	14.2	31.6	0.71	21.0	14.0
FFA-2	–	–	–	–	40.8	0.71	20.7	14.0
OPA	29.9	0.69	20.3	13.5	31.7	0.69	17.5	14.3
PPA	38.8	0.71	20.2	13.8	34.1	0.71	19.0	13.5
RSC	42.0	0.67	22.5	15.5	41.7	0.66	21.2	15.0
OWFA	35.2	0.64	22.0	14.1	33.7	0.67	21.3	14.7
mfs-words	34.6	0.70	21.3	13.6	–	–	–	–
Untuned	30.6	0.76	17.8	11.3				
CLIP	11.7	0.45	18.3	16.4				

## Discussion

We have shown that the image feature representations extracted from convolutional neural networks pretrained on image classification tasks can be made more predictive of fMRI activity in the human visual cortex by fine-tuning using a contrastive learning (CL) approach [30] that maximizes a lower bound on the mutual information [33] between the fMRI activity and the image features. This required a novel adaptation of the SimCLR approach [4] to pair disparate spaces of natural and artificial neural responses, similar to the CLIP model [5] trained to pair images with captions. Essentially, the CNN’s processing is modified such that the representations of images is better aligned to the human subject’s brain’s representation of the same images. We believe this approach is a natural evolution of neural encoding models that seek to capture the dependency of stimulus and neural response [54].

Our approach of fine-tuning ROI- and subject-specific feature extraction models using CL: 1) improves a majority of the voxels for each subject of the voxel’s linear encoding on the Natural Scenes Dataset [3]; 2) exhibits cross-subject transferability via pooling responses from different subjects, especially for early visual ROIs, and enables improvements in encoding for all 9 subjects from another lower-resolution dataset [11]; and 3) reveals divergence from the original CNN in terms of performance and similarity of prediction on downstream tasks across subjects and ROIs with meaningful interpretation of ROI-specific model similarities.

In both early visual and higher visual ROIs our CL approach significantly improves the prediction (in terms of average correlation) versus a pretrained AlexNet baseline for the NSD dataset with a sample size of *n* = 8 (paired, one-tailed t-test with Bonferroni correction for 2 tests and significance threshold of α=0.1). The encoding accuracy, as measured by the average correlation across voxels and averaged across subjects, was improved from 0.386 to 0.409 in the early visual ROIs and from 0.354 to 0.364 in the higher visual ROIs. An average of 92.9% and 77.6% of the voxels for the early and higher visual ROIs, respectively, were improved when compared to the predictions of the baseline pretrained AlexNet (which therefore means worsening the prediction in 7.1% and 22.4% of voxels, respectively).

We also found that fine-tuning networks by directly training a regression head to predict the voxels for each ROI also improves the encoding performance (Neural Network Regression Model), achieving higher average correlations in both the early (0.417) and higher (0.367) ROIs. Yet the CL approach better predicts a majority of voxels in early (61.5%) and higher (57.7%) ROIs as compared to the regression approach. The differential performance coincides with the cost functions: regression achieve higher correlation due to its mean-squared error loss; whereas the CL cost seeks to maximize representational similarity under a cosine similarity that is invariant to the norm of the feature representation. The inclusion of a linear and non-linear projection when forming a representation of the fMRI response in an ROI may filter out some sources of the fMRI variance, causing the image representation to likewise ignore some aspects. Furthermore, the regression-tuning pushes the AlexNet model further away from the original model, as evidenced by the poor classification accuracy of the regression-tuned models on downstream tasks (Fig 6) and separation of the regression-tuned models from the original model in the model landscapes (S8 Fig). A noted difference is that later layers are selected when forming the encoding model for the regression-tuned models (Fig 5), indicating that the whole model is trying to improve the voxel-wise regression. In contrast, the CL approach may be filtering out too much variance, and when fitting encoding models earlier layers are selected (Fig 4). Furthermore, RSA analysis comparing the fMRI-based dissimilarites to model-based dissimilarities shows that after taking the representations from the tuned models at the best layer for encoding, the CL-tuned have higher RSA in early ROIs compared to regression-tuned. However, generally regression tuning creates representations whose dissimilarity better captures the fMRI response. Thus, although both fine-tuning approaches offer improved encoding performance, the way features are extracted is distinct. This identifies the importance of the cost function when seeking brain-aligned image representations.

Importantly, the fMRI responses from different subjects in the same early visual ROIs can be combined to train pooled models which outperform the single-subject models for most (7 out of 8) subjects. When using the pooled approach, the average encoding correlation across subjects is further increased to 0.428 for the early visual ROIs and 0.368 for the higher visual ROIs.

Previous work [55] shows that feature extraction models tuned to the fMRI responses for a specific subject in an early visual ROI can be used in the same ROIs in other subjects with minimal drop in encoding performance, suggesting that the learned feature representations in the fine-tuned AlexNet are common across the different subjects for a particular ROI. Those intersubject results have the same feature extraction models but a more constrained hyper-parameter search (using the layer that originally was best for untuned AlexNet and selecting a single regularization hyper-parameter per ROI, rather than voxel-wise). Specifically, Subjects 3–8 had an equal or higher number of voxels improved using Subject 1’s CL-tuned feature extraction model compared to their own. Likewise, Subject 4, 6, 7, and 8 had a larger number of voxels improved using Subject 2’s CL-tuned model. Not that Subjects 1–2 had the highest noise ceilings [3]. Generally, inter-subject performance was worse for the higher group of ROIs with the exception of Subjects 3 and 8, which had improvements when using Subject 5’s model. These intersubject findings are also confirmed by similarity-based embedding in the model landscapes. Interestingly, the early group of ROI-tuned models from subjects 1 and 2 are more predictive of the responses for all other subjects than those subject’s model tuned specifically on their data. In contrast, for the higher visual and anatomical ROI groups, nearly all of the subjects’ test responses were best predicted by the models tuned on their own training data. Previous work by [56] showed that higher inter-subject variability exists in the structure of higher visual ROIs than ROIs in early visual cortex, which could explain why the subject pooling is more effective for the early visual ROIs.

We further validated our approach by applying the pooled fine-tuned feature extraction models tuned on the NSD dataset to 9 subjects from the Natural Object Dataset [11] for early visual ROIs. Notably, the dataset is lower resolution (3T versus 7T), and we only used 50 voxels per ROI with the highest noise ceilings. We fit new linear models to predict the fMRI responses in NOD using the same feature extraction models as for NSD. We found that an average of 70.6% of voxels were improved versus the untuned AlexNet baseline across all early visual ROIs and subjects when using the CL-tuned models fit on the NSD dataset. With the CL-tuned models the average correlation increased from 0.142 to 0.153, an improvement of 8.1%. The gains in average correlation across subjects ranged from 1.1% to 18.2%. This finding further suggests that the CL fine-tuning on specific ROIs can help in lower resolution datasets. Finally, it confirms that the CL fine-tuned AlexNet backbone extracts visual features more predictive of the early visual ROIs.

Beyond encoding, we used downstream classification tasks to examine the performance of the set of CL-tuned AlexNet models. While all of the brain-tuned models experienced small to moderate decreases in accuracy compared to the untuned (but pretrained on ImageNet) AlexNet, models tuned to higher level ROIs (EBA, OPA, PPA) had minimal drops in accuracy on downstream classification tasks. Notably, the CL-tuned models had much better performance than the regression tuned models. Interestingly, the pooled models had worse downstream performance but exhibit a landscape and relative pattern of performance across ROIs that matched the subject-specific models. This shows that the CL-tuning with more brain data may further deviate from the pretrained AlexNet, yielding better encoding performance (Table 3) but worse downstream task performance. Holistically, across ROIs the CL-tuned models with highest encoding accuracy (pooled early visual) had the lowest downstream classification. This makes sense as the classification tasks require higher-level features. In terms of the similarity-based embedding in the model landscapes, pooled models initially appear distinct from subject-specific models due to the increased dataset size creating further fine-tuning (see S8 Fig); however, examining the pooled and subject-specific models separately reveals that the relative similarity among the ROIs are matched (see Figs 10–12). The two key features of this landscape are that the the CL-tuned models are organized primarily by early to late, with the late models being more similar to untuned, and that the ROIs for the left and right hemispheres of early visual ROIs are separated, converging at later ROIs.

The RSA-based landscape analysis recapitulates this same organization among ROIs. Based on the landscape analysis, whose organization is consistent with semantic similarity among ROI groupings, we would expect some level of transfer between ROIs that are close in these landscapes. To our knowledge this is the first demonstration that the Bhattacharyaa-based dissimilarity measure on softmax predictions [8] is highly correlated with the RSA analysis of the underlying latent embeddings. While this is limited to a 1000-class prediction head and tested on ImageNet, this may be a useful observation.

Finally, as a simple example that highlights the potential for in-silico experimentation with the ROI-specific fine-tuned models, we generated images from Stable Diffusion [17] using the embeddings from the CL-tuned models as prompts. We found that the tuned models for the same ROI in different subjects produced perceptually similar images, while the tuned models for a different ROI produced a set of images with meaningful differences. While this is an interesting approach to understand the ROI-tuned models, other results indicate that Stable Diffusion collapses variation. Another generative method such as diffusion posterior sampling may be necessary. Also, while we used the orthogonal Procrustes method to align the ROI-tuned model to the CLIP embedding, this may not be optimal.

### Limitations

We note that a number of benchmarks were set for encoding on the Natural Scenes Dataset (NSD) [3] based on the Algonauts Project 2023 competition [42]. We do not claim to achieve state of the art, but rather we use NSD as a uniquely large dataset to understand how contrastive learning can yield ROI and/or subject-specific tuning of feature extraction models. Our contribution is a method to compare ROI-specific fine-tuned models by examining their transferability and their similarity and performance on different downstream tasks. Likewise, we have used the Natural Object Dataset [11], which uses a different parcellation scheme [47] than the manual ROI definitions in NSD, to confirm the transferability of the feature extraction models tuned to early visual ROIs which outperform the baseline, but do not directly compare to the previous encoding experiments [11] that used population receptive field models for retinotopic mapping and $\ell_{1}$ regularized linear regression.

The design choices we have used may not be optimal. In particular, using ridge regression is not ideal as it does not account for the correlation of neighboring voxels. Partial least squares or rank constraints across the weight matrices may further improve the encoding [57,58].

The choice of a nonlinear projection head, while used in SimCLR [4], is not used by CLIP [5]. Prior work, [4] and [44], showed that more information may be preserved if a non-linear projection head is used, but then dropped. However, for our purposes the choice of a dimension-reducing linear projection and/or non-linear projection head on the voxel response is questionable since all of the information in the response is relevant. It may be the case that with the linear projection (of reduced dimension) and non-linear projection head the contrastive learning optimizes the model for a subset of the voxels in an ROI that best aligns with the feature extraction. This could explain why the encoding results are not improved across all voxels. For linear encoding, using the neural response and network activations directly (or through a variance preserving projection) may be more optimal. Nonetheless, it is necessary to have at least one linear map so that the fMRI response and AlexNet embedding are of the same dimension. Future work should examine the benefit of dimensionality reduction and/or the non-linear projection head.

Additionally, our choice of scaling the embedding dimension in the contrastive learning loss to be proportional to the voxels in an ROI for the subject-specific ROI-specific models may not be optimal. With the pooled tuning, we tested whether using a constant embedding dimension in the CL loss performs better than an embedding dimension proportional to the number of voxels. As shown in S1 Table, while not significant in terms of encoding performance, the constant embedding dimension had marginally better performance in terms of both average test set correlation and percentage of voxels improved. This means that the particular choice of proportional scaling may not be an optimal hyper-parameter, but is not degrading performance and does not affect any of the conclusions.

Similarly, we have used a fixed temperature for the loss function. Although the temperature is in the range of prior work [4], it may not be optimal and may limit the lower bound estimate of mutual information. In contrast, CLIP [5] treats the temperature as a learnable parameter.

While we tuned a separate model for each ROI and selected a single layer per ROI, it could be argued that one could use a whole brain approach that tunes a single feature extraction model useful for predicting all ROIs. However, it is not clear how the subsequent analysis of this feature encoding would be approached. Instead, we explicitly disentangle the representation by creating a differently fine-tuned model for each ROIs. The benefit of our approach is that we can analyze the ROI-specific models. However, a limitation of our approach is the computation required—which has limited our hyper-parameter searches. By selecting a single layer from the network for the feature extraction of the encoding model, we essentially assume that each ROI is homogeneous in the complexity of the processing. Given the limited granularity of AlexNet layers and relatively fine-grained ROIs, this assumption seems reasonable.

Future work could, firstly, fine-tune the image feature extraction using information pooled from the fMRI responses across different regions of the visual processing stream (i.e., a whole-brain contrastive learning), and secondly, incorporate cross-attention mechanisms to use different ROIs to tune different parts of the CNN, similar to the approach of [29] used to build models predictive of V1-V4, but extended to more ROIs.

More generally, the contrastive learning approach could be used with neural data from other experimental modalities to tune other deep neural network architectures, such as fMRI responses to spoken or read text being used to modify language models. We note that a limitation of our work is that we used a low-capacity network compared to modern architectures. This was necessary since the computational budget of training all subject and ROI specific models would be higher with networks such as ResNet [39] or vision transformers as in CLIP [5]. However, the fact that CL-tuning still selects earlier layers after fine-tuning indicates that the capacity of the network is not limiting for early vision ROIs. Nonetheless, a higher capacity network may be necessary for higher ROIs or for regression tuning. Although we compared results to CLIP, we did not compare to modern pretrained feature extraction networks such as DINOv2 [59].

## Conclusion

In this work, we developed a novel methodology for neural encoding, based on using a contrastive loss to fine-tune a neural network to maximize a lower bound on the shared information between the fMRI responses in visual regions of interest (ROIs) and the stimulus images of natural scenes. We found a significant improvement in encoding performance across both early visual ROIs and in higher visual cortex areas identified as being selective for words, faces, bodies, or places, but with greater improvements in the early ROIs. The representations learned by fine-tuning for individual subjects tend to be transferable as feature extractors in the early visual ROIs, as indicated by the further encoding improvements achieved by training pooled models that combine the fMRI responses from different subjects, and by the improvements over untuned AlexNet for subjects from a separate dataset. We used classification performance of classifiers built on top of fine-tuned models on both ImageNet, the original training task for the untuned AlexNet, and two other image classification datasets (consisting of objects and places), to understand how much the fine-tuned networks deviate from the untuned network. We used the similarity of predictions to create model landscapes to visualize the similarity of responses, revealing multiple axes of dissimilarity associated to early versus late ROIs, lateralization in early ROIs, and separation of place from body and scene areas. We found that pooling together neural responses from different subjects to scale up the training dataset used for fine tuning further improves encoding performance in early visual areas, and creates more deviations from the untuned model, but reveals the same axes of dissimilarity as the subject-specific tuning. Finally, we show how the stark differences of feature extraction from different ROI-specific tuned models and similar subject-specific models can be visualized through image-to-prompt [49] and generative models [17]. Overall, this work points to the potential of aligning different layers of one neural network to different brain regions, and using the brain-tuned models for subsequent in-silico investigations.

## Supporting information

S1 Fig ROI presence table. ROIs present in each subject from NSD, with black indicating that the ROI is present in the subject, and white indicating that the ROI is not present.
(EPS)

S1 Appendix Estimation of mutual information lower bound. (PDF)

S2 Fig Mutual information lower bound estimates. Mutual information lower bound estimates for each subject and ROI, in bits. The rightmost column is an average of the estimates for all subjects which have that ROI. Blank entries indicate that the ROI given by the row is not present in the subject given by the column.
(EPS)

S3 Fig Mutual information lower bound estimates versus ROI size. Lower-bound estimates on MI between the fMRI responses in each ROI and the corresponding ROI-specific CL-fine-tuned AlexNet output versus the average number of voxels in that ROI across subjects. For the subject-specific models the average MI is plotted. For the pooled models the embedding layer dimension is based on the average of the voxel dimensions of all subjects with the ROI. ROI groups are color coded.
(EPS)

S1 Table Pooled results for higher visual ROIs. For each subject, the average test set correlation *r* as described in Accuracy Metric across all higher visual ROIs is reported for CL-tuned AlexNet (Contrastive Learning for ROI-specific Feature Extraction) and pooled CL-tuned models using both a embedding layer dimension equal to the average voxel dimension of all subjects (h-avg) and an embedding layer dimension equal to the maximum voxel dimension over all subjects and ROIs (h-constant) (Pooled Models). The percentage of voxels for which each version of pooled model was more predictive on average across the test set compared to the subject specific model is also shown.
(PDF)

S4 FigRSA between fMRI to model with CLIP.Scatter plot comparing the representational similarity analysis (Spearman’s ρ using either correlation distance (corr) or Euclidean distance (euc) as the dissimilarity measures) between each ROI’s fMRI response and the untuned AlexNet model’s representation, CLIP’s representation, and a CL fine-tuned model. Error bars indicate standard errors across the subjects.(EPS)

S5 Fig Downstream classification performance of ROI-tuned models on Imagenet. Test set accuracies for Imagenet dataset for models for each ROI (both subject-specific and pooled), as well as untuned AlexNet for comparison.
(EPS)

S6 Fig Downstream classification performance of ROI-tuned models on Places365. Test set accuracies for Places365 dataset for models for each ROI (both subject-specific and pooled), as well as untuned AlexNet for comparison.
(EPS)

S7 Fig Downstream classification performance of ROI-tuned models on Caltech256. Test set accuracies for Caltech256 dataset for models for each ROI (both subject-specific and pooled), as well as untuned AlexNet for comparison.
(EPS)

S8 Fig Overall model landscapes. Model landscape using the multidimensional scaling embedding of the Bhattacharya-based dissimilarity for the oracle predictions (large diamond marker), untuned AlexNet (star marker), subject-specific ROI-specific CL fine-tuned models (black borders), subject-pooled ROI-specific fine-tuned models (magenta borders), and subject-specific ROI-specific regression fine-tuned models (cyan borders). Marker face colors indicate classification accuracy clipped to fine-tuned range. Average classification per ROI group and model tuning appears in the legend.
(EPS)
