# Supplementary material for: Contrastive learning to fine-tune feature extraction models for the visual cortex
Source: PLoS Comput Biol. 2026 Aug 17;22(8):e1014656. doi: 10.1371/journal.pcbi.1014656 (PMC13492995; doi:10.1371/journal.pcbi.1014656)
Supplement: Supplementary file 1 [file pcbi.1014656.s002.pdf]

S1 Appendix. Estimation of mutual information lower bound. For continuous random variables $X \in \mathcal{X}$ and $Y \in \mathcal{Y}$ which are jointly distributed, with independent and identically distributed copies of size K, $\{(X_i, Y_i)\}_{i=1}^{K}$, the mutual information between the variables $I(X, Y)$ is lower bounded by the following noise-contrastive estimation (NCE) where $\omega : \mathcal{X} \times \mathcal{Y} \to \mathbb{R}$ is a continuous function [1],

$$I(X,Y) \geq \sup_{\omega} \mathbb{E}[\frac{1}{K}\sum_{i=1}^{K} \log \frac{e^{\omega(X_i,Y_i)}}{\frac{1}{K}\sum_{j=1}^{K} e^{\omega(X_i,Y_j)}}] \tag{1}$$

NCE's lower bound is itself upper-bounded by $\log(K)$, where K is the batch size. Thus, cases of relatively large values of mutual information require a large batch size. Any mutual information estimate is limited and (1) is conservative in practice even when $I(X, Y) \leq \log(K)$ [2].

In the case of neural responses to natural images, the fine-tuned feature extraction model f, the linear mappings to a shared latent space $\mathbf{W}^x, \mathbf{W}^y$, and the non-linear projection head h yield the function $\omega : \mathcal{I} \times \mathcal{Y} \to [0, \frac{1}{\tau}]$

$$\omega(x, \mathbf{y}) = \frac{1}{\tau}\text{sim}(\mathbf{z}^x, \mathbf{z}^y) = \frac{1}{\tau}\text{sim}(h(\mathbf{W}^x f(x)), h(\mathbf{W}^y \mathbf{y})), \tag{2}$$

which can also be interpreted as a non-linear similarity function between the fine-tuned AlexNet activations $\mathbf{a} = f(x)$ and the voxel activations $\mathbf{y}$. Theoretically, the optimal function is $\omega^*(x, \mathbf{y}) = \log p(\mathbf{y}|x) + c(\mathbf{y})$ where $p(\mathbf{y}|x)$ is the conditional density and $c(\mathbf{y})$ is any function that does not depend on x [3]. If ω is close to optimal and $\mathbf{y}$ is the response to image x, then the vectors $\mathbf{z}^x$ and $\mathbf{z}^y$ should have a relatively high cosine similarity (small angle) yielding a relatively high conditional density $\log p(\mathbf{y}|x)$. If x and $\mathbf{y}$ are independent then the vectors should have low cosine similarity on average with low conditional density $\log p(\mathbf{y}|x)$.

After training, the CL loss function enables the computation of a lower bound on mutual information between the image input as represented by the fine-tuned AlexNet and the fMRI activity. While potentially loose [2], we compute estimates of the lower bounds on the mutual information $I(X, Y)$ between the CL-tuned AlexNet representations and the fMRI responses using (1) for all ROIs across all subjects. Fig S2 shows the lower bound estimate for CL-tuned subject-specific and ROI-specific model and CL-tuned ROI-specific models, as well as an average for each ROI over all subjects that have that ROI.

There is a large gap before the theoretical upper bound of this lower bound determined by the batch size ($\log_2(1024) = 10$ bits), and the largest estimate ROI mutual information of 3.78 bits for OPA (left hemisphere) in subject 2. Nonetheless, the relative mutual information across the subjects and ROI specific models shows generally higher information in higher level areas (EBA, OPA, PPA). Some subject showed relatively high mutual information in lower level areas such as V1 and hV4.

Fig S3 shows a plot of the average lower bound estimate across subjects for each ROI versus the average number of voxels in that ROI across the subjects, for both the subject-specific models and the pooled models when using the average embedding layer dimension. Generally, larger ROIs can carry more information, but this saturates above 200 voxels. The pooled models appear to have roughly the same information compared to the subject-specific models.

While increasing mutual information with dimensionality makes sense, it is not clear if the relatively low values of the MI estimate and the saturation effects are due to limitations of the NCE bound, or whether the noise in fMRI causes the information content to be limited. To investigate this we used the classification accuracy on ImageNet's 1000 classes and Fano's inequality [4] to lower bound the mutual

information. Using the accuracy on ImageNet and Fano's inequality the information content related to the class labels of the brain-tuned image feature representation must be higher than the mutual information between the representation and the neural response. For example, with a uniform distribution across ImageNet's 1000 classes Fano's inequality based on the accuracy [4] yields a lower bound of 3.3 bits for subject 1 EBA (left hemisphere), compared to 2 bits from the NCE estimate shown in Fig S2. Thus, future work is needed to explore better mutual information estimation techniques [2] to fMRI responses to stimuli.

References

1. Poole B, Ozair S, Van Den Oord A, Alemi A, Tucker G. On variational bounds of mutual information. In: International Conference on Machine Learning. PMLR; 2019. p. 5171–5180.

2. McAllester D, Stratos K. Formal limitations on the measurement of mutual information. In: International Conference on Artificial Intelligence and Statistics. PMLR; 2020. p. 875–884.

3. Ma Z, Collins M. Noise Contrastive Estimation and Negative Sampling for Conditional Models: Consistency and Statistical Efficiency. In: Proceedings of the 2018 Conference on Empirical Methods in Natural Language Processing; 2018. p. 3698–3707.

4. Scarlett J, Cevher V. An Introductory Guide to Fano's Inequality with Applications in Statistical Estimation. Information-Theoretic Methods in Data Science. 2021; p. 487–528.
