# Supplementary material for: Contrastive learning to fine-tune feature extraction models for the visual cortex
Source: PLoS Comput Biol. 2026 Aug 17;22(8):e1014656. doi: 10.1371/journal.pcbi.1014656 (PMC13492995; doi:10.1371/journal.pcbi.1014656)
Supplement: Supplementary file 2 [file pcbi.1014656.s005.pdf]

S1 Table. Pooled results for higher visual ROIs. For each subject, the average test set correlation r across all higher visual ROIs is reported for CL-tuned AlexNet and pooled CL-tuned models using both a embedding layer dimension equal to the average voxel dimension of all subjects (h-avg) and an embedding layer dimension equal to the maximum voxel dimension over all subjects and ROIs (h-constant). The percentage of voxels for which each version of pooled model was more predictive on average across the test set compared to the subject specific model is also shown.

	**Sub-Specific Avg. $\bar{r}$**	**Pooled (h-avg) Avg. $\bar{r}$**	**Pooled (h-constant) Avg. $\bar{r}$**	**Pooled (h-avg) > Sub-Specific**	**Pooled (h-constant) > Sub-Specific**
1	0.403	0.399	0.400	44.2%	47.6%
2	0.436	0.426	0.427	32.7%	33.8%
3	0.350	0.357	0.359	51.0%	53.9%
4	0.342	0.354	0.356	58.0%	63.5%
5	0.474	0.463	0.465	27.5%	32.8%
6	0.314	0.329	0.330	58.7%	61.1%
7	0.331	0.342	0.342	62.5%	61.4%
8	0.262	0.271	0.274	57.9%	62.4%
	0.364	0.368	0.369	49.1%	52.1%
